# Ets21c Governs Tissue Renewal, Stress Tolerance, and Aging in the *Drosophila* Intestine

**DOI:** 10.1016/j.celrep.2019.05.025

**Published:** 2019-06-04

**Authors:** Juliane Mundorf, Colin D. Donohoe, Colin D. McClure, Tony D. Southall, Mirka Uhlirova

**Affiliations:** 1 Institute for Genetics and Cologne Excellence Cluster on Cellular Stress Responses in Aging-Associated Diseases (CECAD), University of Cologne, Cologne 50931, Germany; 2 Center for Molecular Medicine Cologne, University of Cologne, Cologne 50931, Germany; 3 Department of Life Sciences, Imperial College London, Sir Ernst Chain Building, South Kensington Campus, London SW7 2AZ, UK

**Keywords:** *Drosophila*, stress signaling, regeneration, aging, intestine, stem cells, enterocytes, JNK, transcription factors, Ets21c

## Abstract

Homeostatic renewal and stress-related tissue regeneration rely on stem cell activity, which drives the replacement of damaged cells to maintain tissue integrity and function. The Jun N-terminal kinase (JNK) signaling pathway has been established as a critical regulator of tissue homeostasis both in intestinal stem cells (ISCs) and mature enterocytes (ECs), while its chronic activation has been linked to tissue degeneration and aging. Here, we show that JNK signaling requires the stress-inducible transcription factor Ets21c to promote tissue renewal in *Drosophila*. We demonstrate that Ets21c controls ISC proliferation as well as EC apoptosis through distinct sets of target genes that orchestrate cellular behaviors via intrinsic and non-autonomous signaling mechanisms. While its loss appears dispensable for development and prevents epithelial aging, ISCs and ECs demand Ets21c function to mount cellular responses to oxidative stress. Ets21c thus emerges as a vital regulator of proliferative homeostasis in the midgut and a determinant of the adult healthspan.

## Highlights

• Ets21c acts downstream of JNK to promote epithelial renewal in the adult intestine
• Both stem cell proliferation and apoptosis of enterocytes require Ets21c function
• Ets21c controls cell-type-specific gene expression programs
• Ets21c loss prevents intestinal aging but renders flies sensitive to stress

## Graphical Abstract

**Figure:**
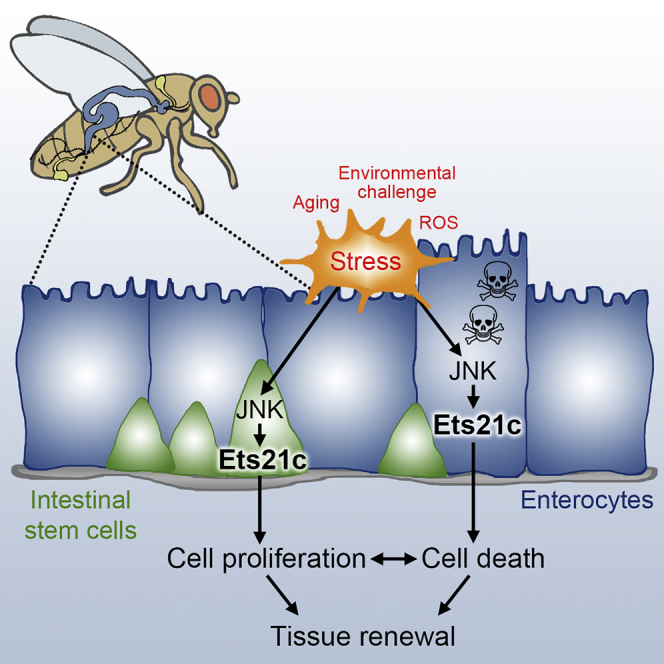

## Teaser

Mundorf et al. show that the conserved JNK signaling pathway requires the transcription factor Ets21c to mount cellular stress responses in the *Drosophila* adult intestine. Ets21c emerges as a critical regulator of tissue homeostasis that coordinates the apoptotic removal of differentiated enterocytes with compensatory stem cell proliferation.

## Introduction

The intestinal epithelium undergoes continuous homeostatic and acute, stress-induced cellular turnover to ensure tissue integrity and function throughout an organism’s lifetime. The replacement of damaged and aberrant cells is fueled by somatic stem cells, whose proliferation is tightly controlled and coordinated with differentiation to satisfy tissue needs while preventing organ degeneration or tumor formation. In the *Drosophila* adult midgut, which is a functional equivalent of the mammalian small intestine, the intestinal stem cells (ISCs) divide asymmetrically to self-renew and generate two different cell types: the transient enteroblasts (EBs) and the enteroendocrine (EE) lineage-determined cells (Biteau and Jasper, 2014, Micchelli and Perrimon, 2006, Sallé et al., 2017, Zeng and Hou, 2015). Following several rounds of endoreplication, the EBs mature into the large, polyploid, and polarized enterocytes (ECs), which represent the major building blocks of the midgut epithelium (Ohlstein and Spradling, 2007). While primarily involved in nutrient resorption, the ECs also serve as a physical and chemical barrier protecting the organism against toxins, pathogens, oxidative stress, and mechanical damage (Buchon et al., 2013). The runaway stem cell activity and loss of intestinal integrity due to chronic inflammation and increased stress load have been recognized as the prime underlying causes of aging-associated tissue decline and lifespan shortening (Biteau et al., 2008, Biteau et al., 2010, Guo et al., 2014). How stress signals are transduced and integrated with the homeostatic maintenance mechanisms at the cellular level and the organ level is only partially understood.

The evolutionarily conserved Jun N-terminal kinase (JNK) signaling is among the key pathways that govern regenerative responses to stress, infection, and damage in the intestine. Its chronic activation has been linked to the breakdown of epithelial integrity and accelerated aging (Biteau et al., 2008, Biteau and Jasper, 2011). JNK signaling affects both ISCs and differentiated ECs. In the ECs, JNK confers stress tolerance and promotes epithelial turnover by triggering the apoptosis of damaged ECs and compensatory ISC proliferation (Apidianakis et al., 2009, Biteau et al., 2008, Buchon et al., 2009, Buchon et al., 2010, Jiang et al., 2009). At the same time, cell-autonomous JNK activation in ISCs accelerates ISC mitosis in cooperation with the epidermal growth factor receptor (EGFR/Ras/ERK) signaling pathway (Biteau and Jasper, 2011, Buchon et al., 2009), which provides the permissive signal for division. In contrast, JNK suppression prevents age-associated ISC hyperproliferation, accumulation of mis-differentiated cells, and epithelial dysplasia, resulting in lifespan extension (Biteau et al., 2008, Biteau et al., 2010, Biteau et al., 2011a). The canonical response to JNK signaling culminates in the activation of transcription factors that orchestrate gene expression. The basic leucine zipper (bZIP) transcription factors Fos (*kayak*) and Jun (*jun-related antigen*) are the best-characterized JNK pathway transcriptional effectors during development (Kockel et al., 2001). In the adult intestine, however, the relation between JNK, Jun, and Fos is less clear. Deficiency for either Fos or Jun interferes with ISC survival, a response that is not observed upon JNK inhibition (Biteau and Jasper, 2011, Buchon et al., 2009). In addition, the transcription factor Foxo has been shown to orchestrate adaptive metabolic responses downstream of JNK in ECs (Karpac et al., 2013). However, Foxo overexpression does not drive epithelial renewal as JNK activation does. These data strongly suggest that other transcription factors may play a role in mediating the pleiotropic, adaptive JNK responses in the different cell types of the intestine.

The transcription factors of the E-twenty six (ETS) family are functionally conserved in all metazoans and are implicated in a plethora of processes, including cell-cycle control, differentiation, proliferation, apoptosis, and tissue remodeling (Hollenhorst et al., 2011, Sharrocks, 2001, Slack et al., 2015). Several genome-wide sequencing approaches have determined that *Drosophila ets21c*, the ortholog of human proto-oncogenes FLI1 and ERG, is transcriptionally induced by infections, wounding, tumorigenesis, and aging (Blanco et al., 2010, Boutros et al., 2002, Broderick et al., 2014, Külshammer et al., 2015, Patterson et al., 2013). In the case of epithelial tumors and lipopolysaccharide treatment, *ets21c* upregulation required JNK activity (Boutros et al., 2002, Külshammer et al., 2015, Toggweiler et al., 2016), thus making Ets21c a plausible candidate to act as a JNK effector in the adult intestine.

Here, we show that Ets21c acts as a critical and specific regulator of ISC and EC functions in the adult fly intestine downstream of JNK signaling and in response to oxidative stress and aging. We demonstrate that Ets21c is necessary and sufficient to coordinate epithelial turnover by controlling ISC proliferation and the removal of ECs. By regulating specific sets of target genes, Ets21c orchestrates distinct cellular behaviors of midgut cells via intrinsic and non-autonomous signaling mechanisms. While dispensable for normal development, Ets21c functions as a vital determinant of stress tolerance and lifespan.

## Results

### Ets21c Functions Downstream of JNK Signaling in the Intestine

**Figure 1:**
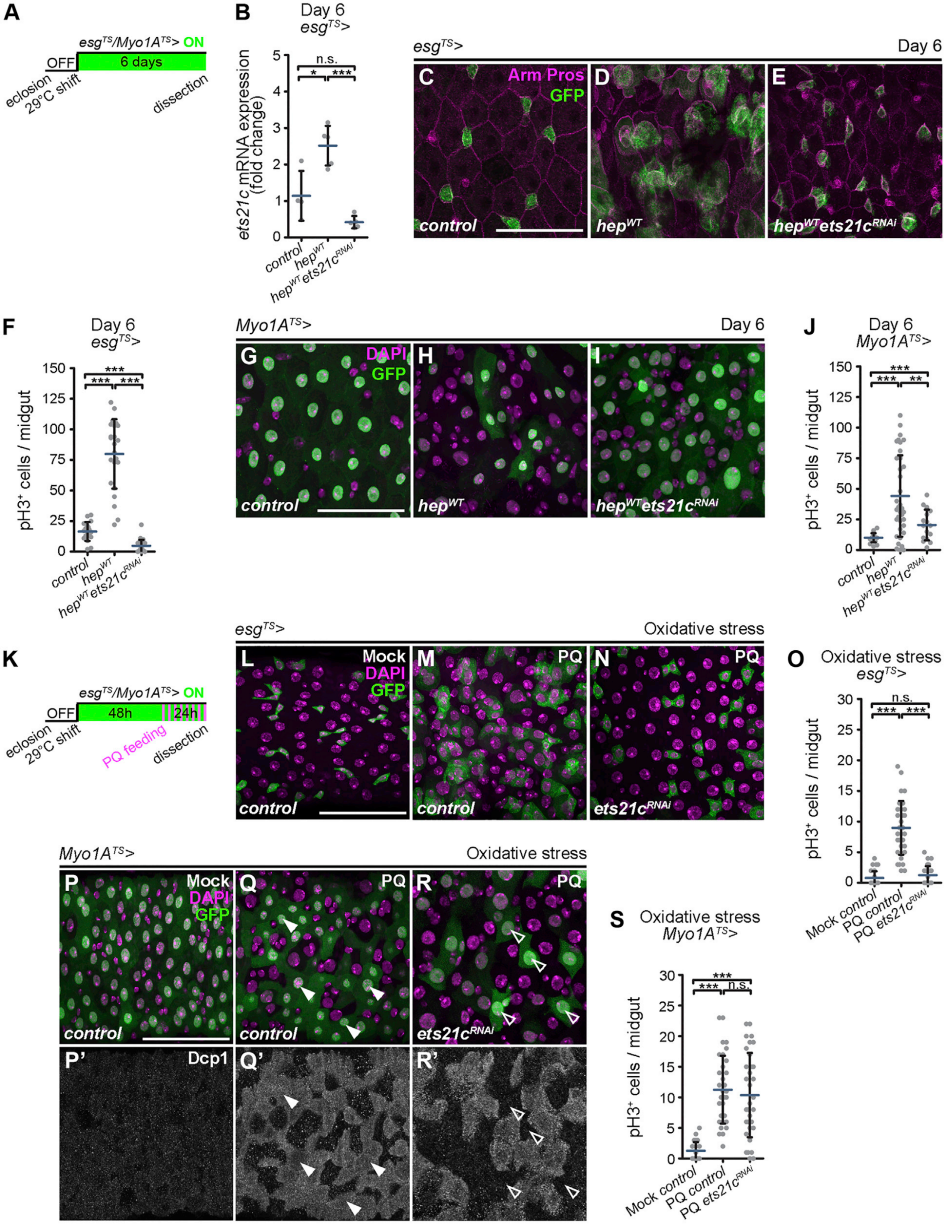
Ets21c Functions Downstream of JNK Signaling in the Intestine (A) Setup and timeline of the TARGET expression system specific for the ISCs/EBs (*esg*^*TS*^) and ECs (*Myo1A*^*TS*^) of adult midguts. (B) qRT-PCR shows *ets21c* expression in 6-day-old midguts overexpressing *hep*^*WT*^ and *ets21c*^*RNAi*^ in ISCs/EBs (*esg*^*TS*^) (n = 4–5). (C–E) Representative confocal images of control 6-day-old posterior midguts (C) and those overexpressing *hep*^*WT*^ only (D) or in combination with *ets21c*^*RNAi*^ (E) in ISCs/EBs (*esg*^*TS*^) marked by GFP. Immunostaining labels cell membranes (Arm) and EEs (Pros). (F) Quantification of pH3^+^ cells per 6-day-old midgut overexpressing *hep*^*WT*^ and *ets21c*^*RNAi*^ in ISCs/EBs (*esg*^*TS*^) (n = 18–23). (G–I) Representative confocal images of control 6-day-old posterior midguts (G) and those overexpressing *hep*^*WT*^ only (H) or in combination with *ets21c*^*RNAi*^ (I) in ECs (*Myo1A*^*TS*^) marked by GFP. DAPI labels nuclei. (J) Quantification of pH3^+^ cells per 6-day-old midgut overexpressing *hep*^*WT*^ and *ets21c*^*RNAi*^ in ECs (*Myo1A*^*TS*^) (n = 16–36). (K) Setup and timeline of stress experiments using paraquat (PQ). (L–N) Representative confocal images of posterior midguts of control flies and those expressing *ets21c*^*RNAi*^ in ISCs/EBs (*esg*^*TS*^) marked by GFP, fed for 24 h with mock solution (L) or 5 mM PQ (M and N). (O) Quantification of pH3^+^ cells per midgut of flies fed for 24 h with 5 mM PQ or mock solution and expressing *ets21c*^*RNAi*^ in ISCs/EBs (*esg*^*TS*^) (n = 34–38). (P–R) Representative confocal images of posterior midguts of control flies and those expressing *ets21c*^*RNAi*^ in ECs (*Myo1A*^*TS*^) marked by GFP, fed for 24 h with mock solution (P) or 5 mM PQ (Q and R). Immunostaining labels activated caspase Dcp1. DAPI labels nuclei. (S) Quantification of pH3^+^ cells per midgut of flies fed for 24 h with 5 mM PQ or mock solution and expressing *ets21c*^*RNAi*^ in ECs (*Myo1A*^*TS*^) (n = 30–33). Data represent means (SDs); ^∗^p < 0.05, ^∗∗^p < 0.01, ^∗∗∗^p < 0.001, n.s. = non-significant. Scale bars: 50 μm. See also Figure S1.

The enhanced expression of *ets21c* in response to diverse stress signals (Blanco et al., 2010, Boutros et al., 2002, Broderick et al., 2014) and its established role downstream of JNK signaling in the *Drosophila* epithelial tumors (Külshammer et al., 2015, Toggweiler et al., 2016) strongly suggest that it may mediate JNK-induced cellular responses in the adult fly intestine. As JNK signaling plays distinct roles in the ISCs and differentiated ECs, we used the temperature-sensitive temporal and regional gene expression targeting (TARGET) expression system (Figure 1A) (McGuire et al., 2004) to manipulate JNK activity in a cell-type-specific manner only during adulthood and test the requirement of Ets21c for the JNK-mediated responses. The specific expression in ISCs and EBs, collectively referred to as progenitor cells (ISCs/EBs), was achieved with the *escargot-Gal4, UAS-GFP, tub-Gal80*^*TS*^ system (hereafter referred to as *esg*^*TS*^) (Micchelli and Perrimon, 2006), while *Myosin 1A-Gal4, UAS-GFP, tub-Gal80*^*TS*^ (hereafter referred to as *Myo1A*^*TS*^) was used to direct transgene expression to the differentiated ECs (Jiang et al., 2009).

We first determined whether JNK signaling is sufficient to regulate *ets21c* expression in the *Drosophila* adult midgut. Moderate activation of JNK signaling in progenitor cells by overexpression of a wild-type form of the JNK kinase Hemipterous (Hep) (*esg*^*TS*^ *> hep*^*WT*^) for 6 days increased *ets21c* mRNA levels (Figure 1B). The JNK-induced *ets21c* transcription was significantly suppressed by RNAi-mediated knockdown of *ets21c* (*esg*^*TS*^ *> hep*^*WT*^*ets21c*^*RNAi*^) (Figure 1B). Immunostaining of the adult guts for the *Drosophila* β-catenin, Armadillo (Arm), revealed the neat organization of the posterior midgut epithelium in young control flies with evenly spaced GFP-expressing progenitor cells (Figures 1C, S1A, and S1E). In contrast, the activation of JNK signaling in ISCs/EBs (*esg*^*TS*^ *> hep*^*WT*^) for 6 days resulted in the accumulation of *esg*^*+*^ cells and a marked distortion of the epithelial architecture (Figures 1D and S1B), as previously reported by Biteau et al. (2008). The increased cell density correlated with enhanced ISC proliferation, as demonstrated by a higher number of phospho-histone H3^+^ (pH3^+^) cells in the whole midgut (Figure 1F). The ISC response to JNK activation was strictly dependent on Ets21c, as ISC overproliferation and tissue dysplasia were suppressed by *ets21c* silencing (*esg*^*TS*^ *> hep*^*WT*^*ets21c*^*RNAi*^) using either of the two independent *ets21c* RNAi lines (Figures 1E, 1F, and S1D).

Activation of JNK in differentiated ECs (*Myo1A*^*TS*^ *> hep*^*WT*^) for 6 days caused a dramatic loss of *Myo1A*^*+*^, GFP^+^ ECs (Figure 1H) compared to the control posterior midguts (Figures 1G and S1J). The JNK-mediated EC loss was, however, compensated for by increased ISC divisions (Figure 1J) and the emergence of *Myo1A*^−^, but polyploid pre-ECs (Figure 1H), as previously reported by Jiang et al. (2009). Simultaneous inhibition of Ets21c in the ECs (*Myo1A*^*TS*^ *> hep*^*WT*^*ets21c*^*RNAi*^) was sufficient to preserve the GFP-expressing ECs (Figure 1I) and reduced the compensatory ISC proliferation (Figure 1J), although the changes to tissue architecture inflicted by EC-specific JNK activation were only partially restored (Figure 1I). Significantly, the requirement for Ets21c in both cell types was specific to JNK but not to the EGFR/Ras/ERK signaling pathway, which provides the permissive signal for ISC division and self-renewal (Biteau and Jasper, 2011, Buchon et al., 2009). Neither gut dysplasia caused by ISC/EB-specific overexpression of a constitutively active form of Ras (*esg*^*TS*^ *> ras*^*V12*^) (Figure S1F) or EGFR (*esg*^*TS*^ *> egfr*^*ACT*^) (Figure S1H) nor excessive EC endoreplication, loss of *Myo1A*^*+*^ ECs, and compensatory ISC proliferation in *Myo1A*^*TS*^ *> ras*^*V12*^ (Figures S1K and S1O) or *Myo1A*^*TS*^ *> egfr*^*ACT*^ guts (Figure S1M) were suppressed by silencing *ets21c* (Figures S1G, S1I, S1L, S1N, and S1O).

To further corroborate the role of Ets21c downstream of JNK signaling, we decided to challenge flies with paraquat, a potent inducer of reactive oxygen species and JNK activity (Biteau et al., 2008, Tang et al., 2013). Feeding flies with paraquat (Figure 1K) caused damage to the intestinal epithelium, resulting in a compensatory ISC proliferation response (Figures 1L, 1M, and 1O) and the activation of the *Drosophila* Death caspase 1 (Dcp1) in ECs (Figures 1P and 1Q). We found that *ets21c* mRNA expression was elevated in the guts of paraquat-treated flies compared to untreated animals (Figure S1P), further supporting the notion that, like JNK (Biteau et al., 2008), Ets21c is involved in mounting the stress response and orchestrating tissue repair. *ets21c* inhibition in progenitors (*esg*^*TS*^ *> ets21c*^*RNAi*^) suppressed the paraquat-induced ISC proliferation (Figure 1O) and expansion of *esg*^*+*^ cells (Figure 1N). Blocking *ets21c* specifically in ECs (*Myo1A*^*TS*^ *> ets21c*^*RNAi*^) of paraquat-exposed flies prevented Dcp1 activation (Figure 1R) and the elimination of mature cells (Figures S1Q and S1R), while the ISC division rate remained increased (Figure 1S), likely contributing to a dramatic swelling of the posterior midgut (Figures S1Q–S1S).

These results implicate Ets21c as a key effector transcription factor, driving responses to activated JNK but not EGFR/Ras/ERK signaling in the adult fly gut. *ets21c* mediates JNK functions in both ISCs and ECs by controlling the proliferation rate of stem cells and elimination of differentiated cells, which are both required for an efficient tissue response to oxidative stress.

### Ets21c Regulates Stem Cell Proliferation, Epithelial Turnover, and Tissue Aging

**Figure 2:**
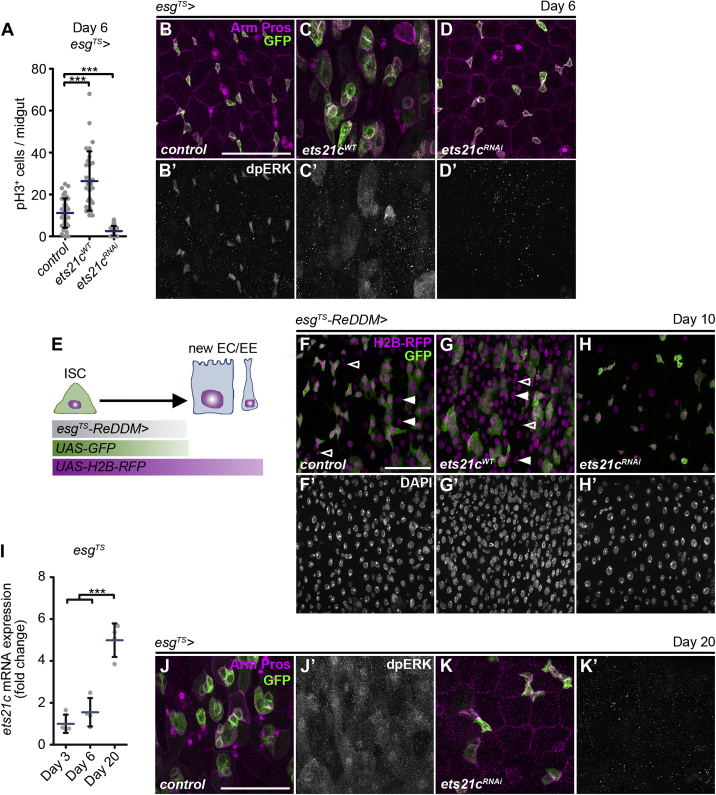
Ets21c Regulates Stem Cell Proliferation, Epithelial Turnover, and Tissue Aging (A) Quantification of pH3^+^ cells per midgut overexpressing *ets21c*^*WT*^ and *ets21c*^*RNAi*^ in ISCs/EBs (*esg*^*TS*^) (n = 30–41). (B–D) Representative confocal images of 6-day-old control posterior midguts (B) and those overexpressing *ets21c*^*WT*^ (C) and *ets21c*^*RNAi*^ (D) in ISCs/EBs (*esg*^*TS*^) marked by GFP. Immunostaining labels cell membranes (Arm), EEs (Pros), and ERK activation (dpERK). (E) Schematic representation of the *esg*^*TS*^*-ReDDM* tracing system. (F–H) Representative confocal images of 10-day-old control posterior midguts (F) and those overexpressing *ets21c*^*WT*^ (G) and *ets21c*^*RNAi*^ (H) in ISCs/EBs with the *esg*^*TS*^*-ReDDM* tracing system. ISCs/EBs are double positive for GFP and H2B-RFP. Newly generated diploid EEs (empty arrowhead) or polyploid ECs (filled arrowhead) are only labeled by H2B-RFP. DAPI labels nuclei. (I) qRT-PCR shows *ets21c* expression in 3-, 6-, and 20-day-old midguts of *esg*^*TS*^ flies (n = 4). (J and K) Representative confocal images of 20-day-old control posterior midguts (J) and those expressing *ets21c*^*RNAi*^ (K) in ISCs/EBs (*esg*^*TS*^) marked by GFP. Immunostaining labels cell membranes (Arm), EEs (Pros), and ERK activation (dpERK). Data represent means (SDs); ^∗∗∗^p < 0.001. Scale bars: 50 μm.

The epistatic relation between Ets21c and JNK in the fly intestine and its involvement in stress-induced regenerative response prompted us to determine the physiological role of Ets21c in the progenitor cells of the midgut. The number of mitotically active cells in the midgut correlated with *ets21c* levels. While ISC/EB-specific *ets21c* knockdown (*esg*^*TS*^ *> ets21c*^*RNAi*^) decreased the ISC division rate (Figure 2A), *ets21c* overexpression (*esg*^*TS*^ *> ets21c*^*WT*^) was sufficient to accelerate ISC proliferation (Figure 2A), resulting in an expansion of GFP-expressing cells and gut remodeling (Figure 2C) as compared to *esg*^*TS*^ control midguts (Figures 2A and 2B). The ISC division rate correlated with ERK activity assessed by the immunostaining against the double phosphorylated ERK kinase (dpERK) (Figures 2B’–2D’). Ets21c, however, appeared dispensable for the maintenance of the identity of ISCs and their survival, as progenitor cells were still present after 6 and 20 days of ISC/EB-specific Ets21c inhibition (*esg*^*TS*^ *> ets21c*^*RNAi*^) (Figures 2D, 2K, and S1C). Thus, consistent with the role of JNK (Biteau and Jasper, 2011), Ets21c is not required for ISC maintenance, but it does control their mitotic rate.

To determine whether the Ets21c-induced changes to ISC mitotic activity affected epithelial turnover, we used the *esg*^*TS*^*-ReDDM* (repressible dual differential stability cell marker) tracing technique (Antonello et al., 2015). In addition to labeling ISCs and EBs with a short-lived mCD8-GFP, the *esg*^*TS*^*-ReDDM* system allows the expression of a long-lived histone H2B-red fluorescent protein (RFP), which marks all progeny derived from the GFP-RFP-double positive progenitor population (Figure 2E). After 10 days, the posterior midgut of control flies partially renewed, being made up of GFP-RFP-double positive ISCs/EBs and newly differentiated RFP^+^ diploid EEs and polyploid ECs next to old unlabeled cells (Figure 2F). In contrast, cell replenishment was far more extensive following *ets21c* overexpression (*esg*^*TS*^*-ReDDM > ets21c*^*WT*^), resulting in a midgut epithelium containing mostly newly generated di- and polyploid RFP^+^ cells (Figure 2G). On the other hand, inhibition of *ets21c* (*esg*^*TS*^*-ReDDM > ets21c*^*RNAi*^) slowed down the steady-state epithelial turnover manifested by the reduced number of RFP^+^ cells in the epithelium (Figure 2H). These data show that Ets21c is necessary and sufficient to promote epithelial renewal by controlling ISC proliferation.

It has been well established that the capacity to coordinate ISC proliferation with epithelial renewal declines with age. In the guts of old flies, JNK activity rises and drives ISC hyperproliferation and accumulation of mis-differentiated cells, causing epithelial dysplasia and breakdown of epithelial integrity (Biteau et al., 2008, Biteau et al., 2010). Consistently, we observed a marked increase in *ets21c* mRNA levels in the guts of 20-day-old relative to 6-day-old flies (Figure 2I), implicating Ets21c function in the age-related changes of the intestinal tissue. Importantly, progenitor-specific *ets21c* inhibition (*esg*^*TS*^ *> ets21c*^*RNAi*^) was sufficient to suppress the aging-associated accumulation of *esg*^*+*^ cells, dpERK enrichment, and tissue dysplasia (Figures 2J and 2K).

Based on our data, we propose that transient upregulation of Ets21c in the stem cell compartment may be essential to confer tissue plasticity, allowing efficient epithelial renewal under stress conditions in particular. However, the Ets21c levels must be tightly controlled as its chronic activation contributes to intestinal tissue aging and dysplasia.

### Ets21c Drives Epithelial Renewal by Triggering EC Apoptosis

**Figure 3:**
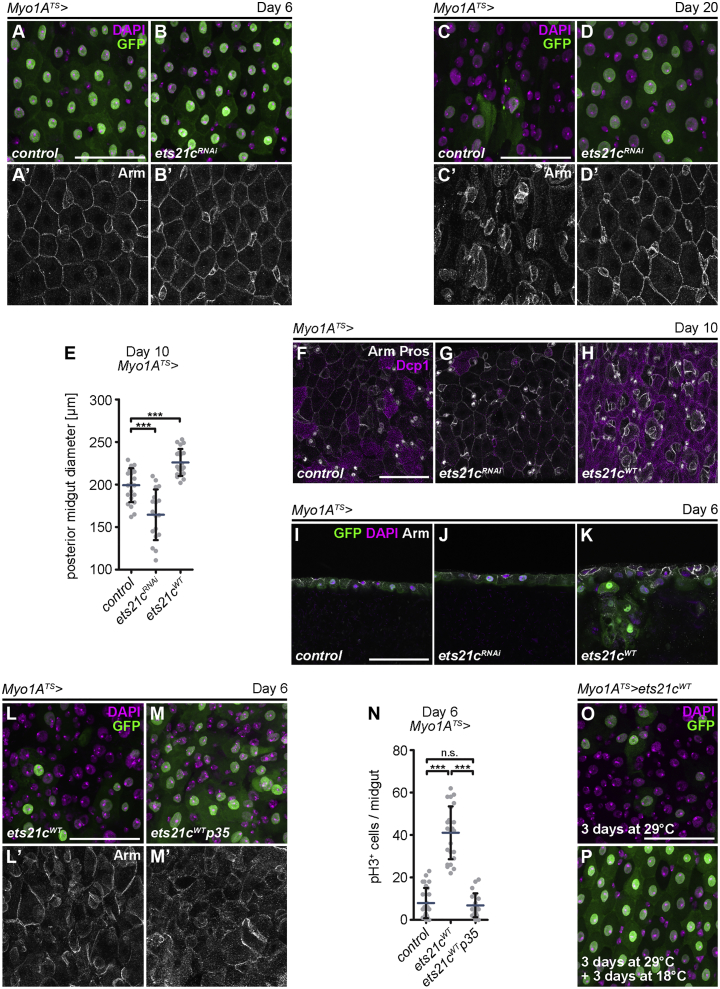
Ets21c Drives Epithelial Renewal by Triggering EC Apoptosis (A–D) Representative confocal images of 6- and 20-day-old control posterior midguts (A and C) and those expressing *ets21c*^*RNAi*^ (B and D) in ECs (*Myo1A*^*TS*^) marked by GFP. Immunostaining labels cell membranes (Arm). DAPI labels nuclei. (E) Diameter measurements of 10-day-old posterior midguts expressing *ets21c*^*WT*^ and *ets21c*^*RNAi*^ in ECs (*Myo1A*^*TS*^) (n = 18–22). (F–H) Representative confocal images of 10-day-old control posterior midguts (F) and those overexpressing *ets21c*^*RNAi*^ (G) and *ets21c*^*WT*^ (H) in ECs (*Myo1A*^*TS*^). Immunostaining labels activated caspase Dcp1, cell membranes (Arm), and EEs (Pros). (I–K) Representative confocal images of 6-day-old control posterior midguts (I) and those overexpressing *ets21c*^*RNAi*^ (J) and *ets21c*^*WT*^ (K) in ECs (*Myo1A*^*TS*^) marked by GFP. Images show single confocal sections of epithelial cross-sections with the gut lumen oriented to the bottom. Immunostaining labels cell membranes (Arm). DAPI labels nuclei. (L and M) Representative confocal images of 6-day-old posterior midguts overexpressing *ets21c*^*WT*^ alone (L) or in combination with *p35* (M) in ECs (*Myo1A*^*TS*^) marked by GFP. Immunostaining labels cell membranes (Arm). DAPI stains nuclei. (N) Quantification of pH3^+^ cells per midgut overexpressing *ets21c*^*WT*^ and *p35* in ECs (*Myo1A*^*TS*^) (n = 20–28). (O and P) Representative confocal images of recovery experiments showing posterior midguts of flies overexpressing *ets21c*^*WT*^ in ECs (*Myo1A*^*TS*^) marked by GFP for 3 days at a permissive temperature (29°C) (O) compared to those shifted back to a restrictive temperature (18°C) for 3 additional days (P). Data represent means (SDs); ^∗∗∗^p < 0.001; n.s. = non-significant. Scale bars: 50 μm. See also Figure S2.

It is important to note that ECs rather than progenitor cells are the first to encounter environmental challenges, given their contact with the hostile luminal milieu. Accordingly, JNK signaling is primarily activated in ECs in response to challenges such as oxidative stress or bacterial infection (Biteau et al., 2008, Buchon et al., 2009). It is therefore conceivable that ECs are the chief cells in which Ets21c-mediated responses would be mounted. This notion is further supported by our observation that paraquat-induced cell death requires Ets21c in ECs (Figure 1R). Thus, we tested Ets21c function in the ECs of young and old flies. As anticipated, the knockdown of *ets21c* in the ECs (*Myo1A*^*TS*^ *> ets21c*^*RNAi*^) of 6-day-old flies when stress load was limited, did not affect the midgut epithelium (Figures 3A and 3B). In 20-day-old guts, however, blocking *ets21c* prevented aging-associated loss of *Myo1A*^*+*^ ECs and gut remodeling (Figures 3C and 3D). Closer examination of flies aged for 10 days revealed that the *Myo1A*^*TS*^ *> ets21c*^*RNAi*^ guts were much thinner (Figure 3E), containing only a few Dcp1^+^ or TUNEL^+^ cells (Figures 3G and S2B) compared to control midguts (Figures 3E, 3F, and S2A).

To determine whether EC-specific Ets21c upregulation would be sufficient to induce epithelial turnover by promoting caspase activation and apoptotic removal of ECs, we carried out chronic and transient overexpression experiments to mimic the conditions of aging and acute stress followed by recovery, respectively. Analysis of *Myo1A*^*TS*^ *> ets21c*^*WT*^ midguts in which Ets21c was chronically activated for 6 and 10 days revealed a marked increase in caspase activity (Figure 3H), TUNEL^+^ cells (Figure S2C), and loss of differentiated *Myo1A*^*+*^ ECs (Figures 3L and S2C). Tissue cross-sections further showed that in contrast to the intact epithelial structure of control and *Myo1A*^*TS*^ *> ets21c*^*RNAi*^ midguts (Figures 3I and 3J), ECs overexpressing *ets21c* (*Myo1A*^*TS*^ *> ets21c*^*WT*^) underwent luminal extrusion (Figure 3K). The dramatic loss of differentiated ECs was accompanied by increased ISC proliferation (Figure 3N), appearance of polyploid but *Myo1A*^*−*^ immature pre-ECs (Figures 3L and S2C), and broadening of the posterior midgut diameter (Figure 3E). To prove that the removal of GFP-expressing ECs upon *ets21c*^*WT*^ overexpression is apoptosis dependent, we co-expressed the pan-caspase inhibitor p35 to block effector caspases. The expression of *p35* (*Myo1A*^*TS*^ *> ets21c*^*WT*^*p35*) counteracted the loss of mature ECs (Figure 3M) and reduced the compensatory proliferation response to *ets21c*^*WT*^ overexpression (Figure 3N). However, the epithelial structure remained disturbed (Figure 3M’), indicating additional functions of Ets21c apart from the regulation of cell death. The loss of EC and changes to the tissue architecture were already apparent after a transient 3-day-long stimulation of Ets21c activity (*Myo1A*^*TS*^ *> ets21c*^*WT*^) (Figure 3O). However, when flies were transferred back to the restrictive temperature (18°C) for another 72 h, mimicking an acute but timely restricted stress, the epithelium regained its normal control-like appearance (Figure 3P).

These data demonstrate that increased Ets21c activity inflicted either by age or oxidative stress affects ECs, and it does so by controlling EC apoptosis, inducing ISC division, and producing pre-ECs, which are essential processes to allow cell replacement. As the Ets21c-mediated responses are inducible and reversible, Ets21c emerges as a critical player in tissue regeneration triggered by acute challenges. The Ets21c activity, however, must be tightly controlled to prevent tissue overgrowth and premature aging. The “young” appearance of aged *Myo1A*^*TS*^ *> ets21c*^*RNAi*^ guts, however, is likely a consequence of reduced epithelial turnover due to restricted removal of mature ECs, a phenomenon that has been previously reported by Takeishi et al. (2013).

### Ets21c Binds to Actively Transcribed Genes as well as Those Devoid of PolII Binding

To determine how Ets21c regulates the observed processes in the different cell types of the gut, we exploited a cell-type-specific genome-wide *in vivo* Targeted DamID (TaDa) mapping approach (Southall et al., 2013) to identify putative direct targets of Ets21c. To this end, we expressed a wild-type Ets21c protein fused to bacterial Dam methylase (*ets21c*^*DAM*^) in ISCs/EBs and ECs using the *esg*^*TS*^ and *Myo1A*^*TS*^ systems, respectively. To determine the transcriptional state of the different cell types, we included samples in which a Dam-polymerase II-fusion protein (*PolII*^*DAM*^) was expressed in either ISCs/EBs or ECs. Of the 5,184 genes associated with Ets21c binding in the ISCs/EBs, 30% were actively transcribed (Figure S3A; Table S2). In the *Myo1A*^*TS*^ samples, Ets21c bound to the proximity of 4,854 genes, while only 12% of them were also bound by PolII (Figure S3B; Table S2). Thus, in young unstressed midguts, Ets21c contacted regulatory loci of actively transcribed (71% of ISC/EB-expressed transcripts and 64% of EC-expressed transcripts) and non-expressed genes.

**Figure 4:**
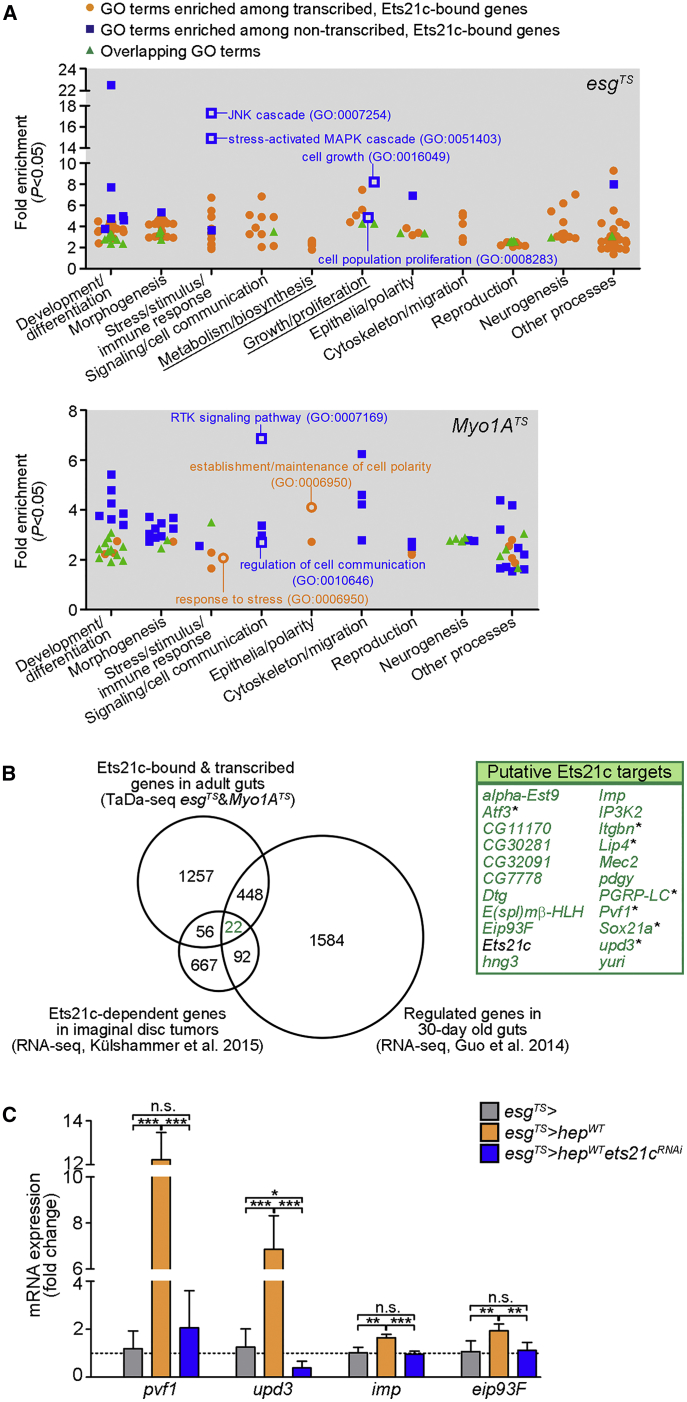
Ets21c Binds to Actively Transcribed Genes and Those Devoid of PolII Binding (A) GO analysis of gene loci occupied by Ets21c and PolII in *esg*^*TS*^*-* and *Myo1A*^*TS*^-expressing adult midgut cells identified by TaDa (see also Tables S2 and S3). Plots depict functional GO terms enriched among the top 250 candidate genes bound by Ets21c. Color coding represents the transcriptional activity based on PolII binding. Each dot represents a single GO term with fold enrichment ≥2 and p < 0.05. Underlined GO groups were overrepresented in *esg*^*TS*^ but not *Myo1A*^*TS*^ samples. (B) Venn diagram shows 22 putative Ets21c target genes that overlap between the Ets21c-TaDa dataset of adult midguts and the transcriptomes of *Drosophila* tumors and aging guts (see also Table S4). Stars indicate genes with reported function in the intestine. (C) qRT-PCR shows the expression of putative Ets21c target genes *pvf1*, *upd3*, *imp*, and *eip93F* in 6-day-old midguts overexpressing *hep*^*WT*^ and *ets21c*^*RNAi*^ in ISCs/EBs (*esg*^*TS*^). Data represent means (SDs); n = 4–5; ^∗^p < 0.05, ^∗∗^p < 0.01, ^∗∗∗^p < 0.001, n.s. = non-significant. See also Figure S3 and Tables S2, S3, and S4.

A Gene Ontology (GO) clustering analysis of the top 250 genes bound by Ets21c that were either actively transcribed or devoid of PolII binding in the different gut cell types (Table S3) revealed an overrepresentation of GO terms associated with basic biological functions ranging from developmental processes to epithelial functions (Figure 4A; Table S3). The set of non-transcribed, Ets21c-bound genes in ISCs/EBs showed enrichment for GO terms linked to “cell growth” and “cell proliferation,” but also “JNK and stress-activated mitogen-activated protein kinase (MAPK) cascades” (Figure 4A; Table S3). These results suggest that Ets21c could prime loci for a fast response to stress and JNK activation. qRT-PCR results supported this notion as the proper expression of several JNK-regulated, cytoprotective, and oxidative-stress response genes (Biteau et al., 2010, Wang et al., 2003, Wu et al., 2009) that showed Ets21c^DAM^ binding (Table S2) required Ets21c (Figure S3C). Also in the *Myo1A*^*TS*^ TaDa samples, the enriched GO categories, including “response to stress,” “receptor tyrosine kinase (RTK) signaling pathway,” “regulation of cell communication,” and “maintenance of cell polarity” correlated with our functional *in vivo* analysis, further emphasizing the role of Ets21c in controlling epithelial remodeling and compensatory signaling (Figure 4A).

### Cell-Type-Specific Sets of Target Genes Mediate the Cellular Responses to Ets21c

Our TaDa approach identified 1,783 genes that were actively transcribed and bound by Ets21c in ISCs/EBs or ECs (Table S2). To select candidates that may be essential for Ets21c-mediated responses, we compared the TaDa results with other available genome-wide sequencing datasets. We considered transcripts that were aberrantly expressed in clonal *ras*^*V12*^*scrib*^*1*^ tumors in the eye-antennal imaginal discs but normalized by *ets21c* silencing (Külshammer et al., 2015) and genes that were regulated in aging 30-day-old versus young 2-day-old gut epithelia (Guo et al., 2014) (Table S4). The overlap of all 3 datasets highlighted 22 genes that could be direct Ets21c effectors in the adult intestine (Figure 4B; Table S4). To functionally validate our genomic approach, we focused on 4 of the putative Ets21c targets: *unpaired 3* (*upd3*), *platelet-derived growth factor (PDGF)- and vascular endothelial growth factor (VEGF)-related factor 1* (*pvf1*), *insulin growth factor-II (IGF-II) mRNA-binding protein* (*imp*), and *ecdysone-inducible protein 93F* (*eip93F*). All four genes were transcriptionally induced by JNK in ISCs/EBs (*esg*^*TS*^ *> hep*^*WT*^), and in line with the TaDa data (Figure S3D), this upregulation required Ets21c function (Figure 4C).

**Figure 5:**
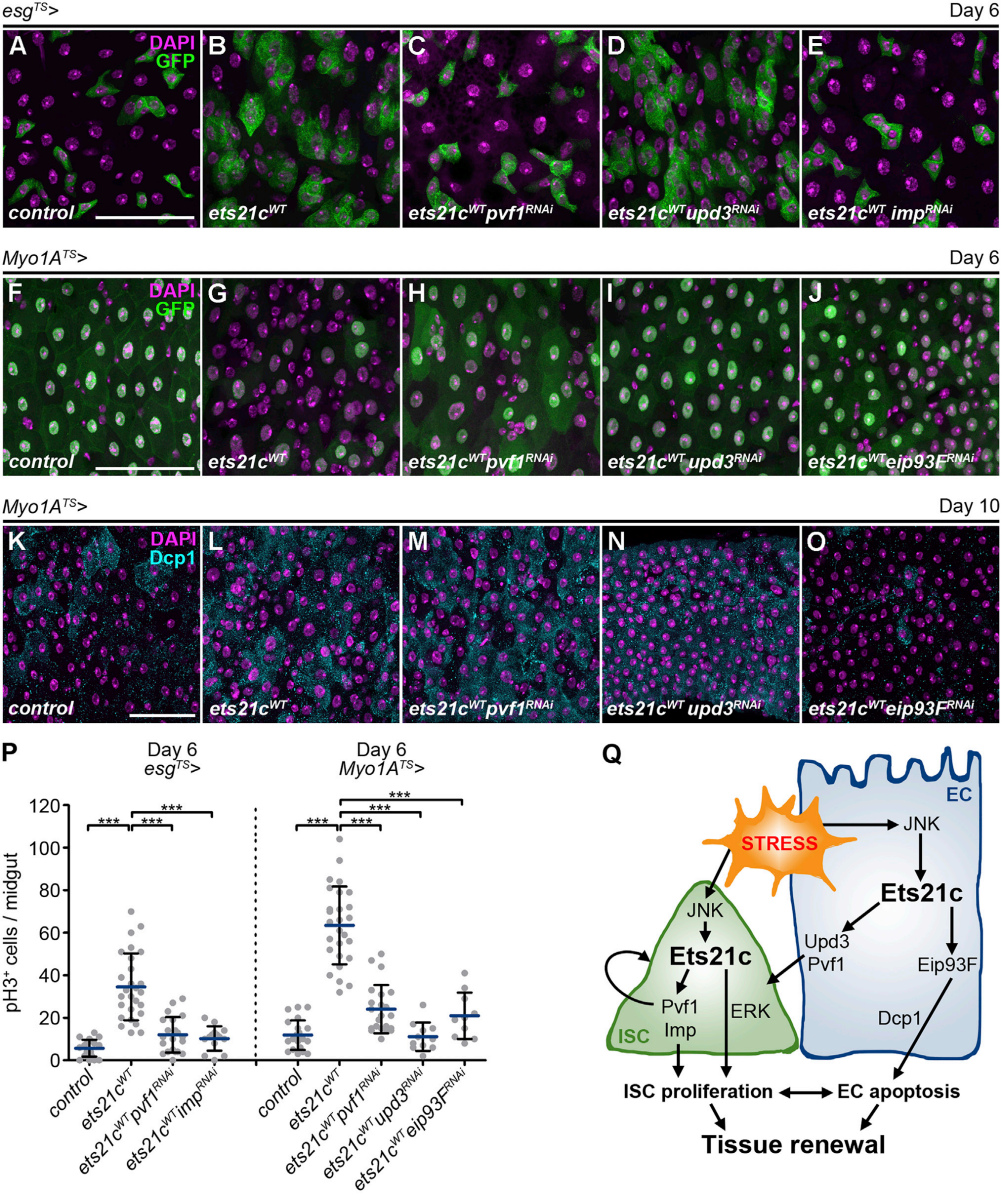
Cell-Type-Specific Sets of Target Genes Mediate the Cellular Responses to Ets21c (A–E) Representative confocal images of 6-day-old control posterior midguts (A) and those overexpressing *ets21c*^*WT*^ alone (B) or in combination with RNAi-transgenes against *pvf1* (C), *upd3* (D), and *imp* (E) in ISCs/EBs (*esg*^*TS*^) marked by GFP. (F–J) Representative confocal images of 6-day-old control posterior midguts (F) and those overexpressing *ets21c*^*WT*^ alone (G) or in combination with RNAi-transgenes against *pvf1* (H), *upd3* (I), and *eip93F* (J) in ECs (*Myo1A*^*TS*^) marked by GFP. (K–O) Representative confocal images of 10-day-old control posterior midguts (K) and those overexpressing *ets21c*^*WT*^ alone (L) or in combination with RNAi-transgenes against *pvf1* (M), *upd3* (N), and *eip93F* (O) in ECs (*Myo1A*^*TS*^). Immunostaining labels activated caspase Dcp1. (P) Quantification of pH3^+^ cells per midgut overexpressing *ets21c*^*WT*^ and RNAi-transgenes against *pvf1*, *upd3*, *imp*, and *eip93F* in ISCs/EBs (*esg*^*TS*^) or ECs (*Myo1A*^*TS*^) (n = 10–27). Data represent means (SDs); ^∗∗∗^p < 0.001. (Q) Schematic representation of Ets21c functions in the *Drosophila* adult intestine. DAPI labels nuclei. Scale bars: 50 μm. See also Figure S4.

To test the requirement of the selected candidates (Upd3, Pvf1, Imp, and Eip93F) for Ets21c-mediated cell-type-specific responses in the intestine, we silenced their function using specific RNAi lines (Figures S4A–S4C; Pahl et al. 2019) in either progenitor cells or differentiated ECs overexpressing *ets21c*. Single knockdown of any of these genes in ISCs/EBs (Figures S4E–S4G) or ECs (Figures S4I–S4K) did not cause any obvious phenotypes compared to 6-day-old control midguts (Figures 5A, 5F, S4D, and S4H), supporting the notion that they may act as mediators of stress-Ets21c-induced responses.

Pvf ligands, which act through binding to the PDGF/VEGF receptor (Pvr), are secreted factors involved in age- and oxidative stress-related responses in the posterior midgut (Bond and Foley, 2012, Choi et al., 2008). Silencing *pvf1* either in progenitors (*esg*^*TS*^ *> ets21c*^*WT*^*pvf1*^*RNAi*^) (Figure 5C) or differentiated ECs (*Myo1A*^*TS*^ *> ets21c*^*WT*^*pvf1*^*RNAi*^) (Figure 5H) reduced the Ets21c-driven epithelial remodeling (Figures 5B and 5G). In addition, Pvf1 inhibition decreased Ets21c-promoted ISC proliferation intrinsically in ISCs/EBs and an EC-induced compensatory proliferation response (Figure 5P). These data suggest that Ets21c engages in the regulation of Pvf1/Pvr signaling to control ISC proliferation in an autocrine as well as a paracrine manner.

Unpaired (Upd) cytokines play an essential role in stem cell maintenance, proliferation, and intestinal turnover under homeostatic and stress conditions affecting tissue aging (Beebe et al., 2010, Jiang et al., 2009). While Upd1 stimulates ISC division in an autocrine manner, Upd2 and Upd3 act non-autonomously, being secreted by damaged ECs (Osman et al., 2012). Consistently, silencing *upd3* in *ets21c-*overexpressing ECs (*Myo1A*^*TS*^ *> ets21c*^*WT*^*upd3*^*RNAi*^) suppressed epithelial dysplasia (Figure 5I) and the ISC mitotic response (Figure 5P), while its knockdown in the progenitors had no effect (*esg*^*TS*^ *> ets21c*^*WT*^*upd3*^*RNAi*^) (Figure 5D). In contrast, inhibition of Imp, which has been shown to control germline stem cell behavior through the regulation of *upd1* (Toledano et al., 2012), prevented *ets21c-*driven ISC proliferation only when silenced in progenitors (*esg*^*TS*^ *> ets21c*^*WT*^*imp*^*RNAi*^) (Figures 5E and 5P) but not in ECs (Figure S4M). These data thus establish an intrinsic ISC requirement for Imp and paracrine Upd3 activity in driving ISC proliferation downstream of Ets21c. In addition, the cell-type-specific suppression of *ets21c* gain-of-function phenotypes by *imp*^*RNAi*^ in ISCs/EBs and *upd3*^*RNAi*^ only in ECs demonstrates that the rescue effects are not a consequence of Gal4/UAS system saturation with multiple transgenes.

Finally, the Ets21c requirement for EC removal prompted our interest in Eip93F, the Pipsqueak family transcription factor, which is known to regulate cell death during fly metamorphosis (Liu et al., 2014). Blocking Eip93F function in ECs overexpressing *ets21c* (*Myo1A*^*TS*^ *> ets21c*^*WT*^*eip93F*^*RNAi*^) reduced EC loss (Figure 5J) and the associated compensatory proliferation response (Figure 5P). Furthermore, the inhibition of Eip93F but not Pvf1 or Upd3 abolished Dcp1 activation, which is characteristic for *ets21c* overexpressing ECs (Figures 5K–5O). The absence of Dcp1 signal also in 10-day-old *Myo1A*^*TS*^ *> eip93F*^*RNAi*^ (Figure S4O) compared to control midguts (Figure S4N) strongly argues for a role for Eip93F in controlling adult midgut epithelial turnover by regulating caspase activity.

Our functional *in vivo* analysis validated four of the putative Ets21c targets and revealed their specific requirements for the distinct Ets21c functions in the ISCs and differentiated ECs (Figure 5Q). Our data show that the Ets21c-mediated responses result from the coordinated regulation of gene expression and interplay between intrinsic and non-autonomous signaling.

### Loss of *ets21c* Prolongs Lifespan but Renders Flies Sensitive to Stress

**Figure 6:**
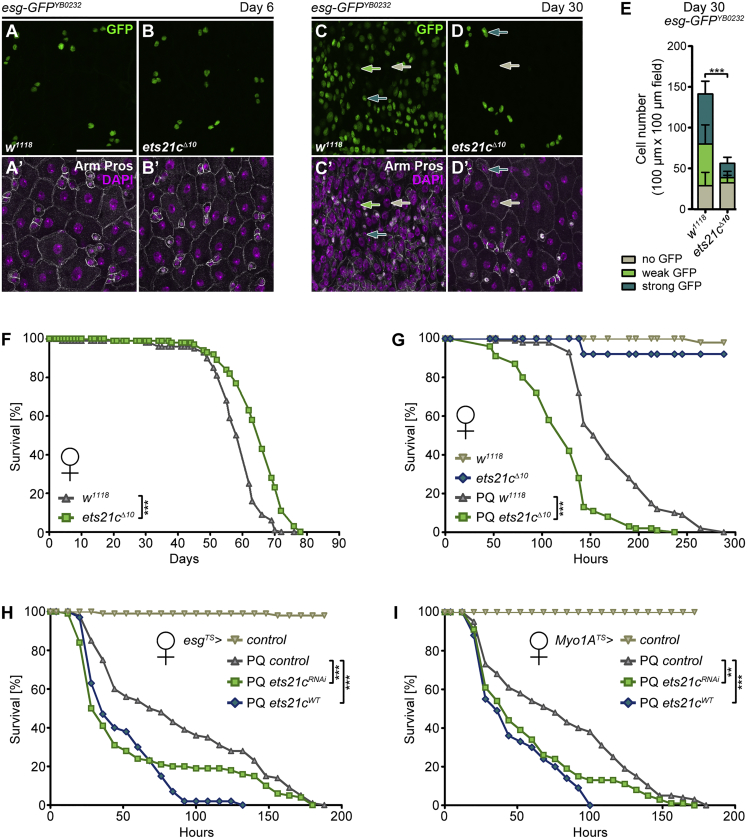
Loss of *ets21c* Prolongs Lifespan but Renders Flies Sensitive to Stress (A–D) Representative confocal images of 6- and 30-day-old *w*^*1118*^ (A and C) and *ets21c*^*Δ10*^ posterior midguts (B and D). ISCs/EBs are labeled with an *esg-GFP*^*YB0232*^ protein-trap reporter. Arrows indicate cells with strong (ISC/EB, blue), weak (pre-EC, green), or no (EC/EE, gray) *esg-GFP*^*YB0232*^ signal in aging midguts. Immunostainings label cell membranes (Arm) and EEs (Pros). DAPI stains nuclei. (E) Quantification of cells per confocal image field that shows strong (ISC/EB, blue), weak (pre-EC, green), or no (EC/EE, gray) *esg-GFP*^*YB0232*^ expression in 30-day-old *w*^*1118*^ and *ets21c*^*Δ10*^ posterior midguts. Data represent means (SDs); n = 13–17; ^∗∗∗^p < 0.0001. (F) Percentage of survival of *w*^*1118*^ (n = 200) and homozygous *ets21c*^*Δ10*^ (n = 314) adult females over time (mean difference of 4 days). (G) Percentage of survival of *w*^*1118*^ (n = 100) and homozygous *ets21c*^*Δ10*^ (n = 120) adult females fed with 5 mM PQ (mean difference of 52 h) or mock solution (*w*^*1118*^ n = 40; *ets21c*^*Δ10*^ n = 60). (H) Percentage of survival of adult females overexpressing *ets21c*^*WT*^ (n = 60; mean difference of 36 h) and *ets21c*^*RNAi*^ (n = 80; mean difference of 31 h) in ISCs/EBs (*esg*^*TS*^) compared to control flies fed with PQ (n = 80) or mock solution (n = 80). (I) Percentage of survival of adult females overexpressing *ets21c*^*WT*^ (n = 80; mean difference of 31 h) and *ets21c*^*RNAi*^ (n = 80; mean difference of 23 h) in ECs (*Myo1A*^*TS*^) compared to control flies fed with PQ (n = 80) or mock solution (n = 40). Lifespan and survival curves represent one of two to three independent experiments. Statistical significance was determined by log rank test; ^∗∗^p < 0.01, ^∗∗∗^p < 0.001. Scale bars: 50 μm. See also Figure S5.

Maintaining intestinal integrity has emerged as an important determinant of health and lifespan. Having established the Ets21c requirement for controlling ISC division rate and death of ECs, we asked whether *ets21c* deficiency would affect fly longevity and survival under stress. Using CRISPR-Cas9 genome editing, we generated a loss-of-function mutant *ets21c*^*Δ10*^ allele. The 10-bp deletion induced a frameshift, resulting in a premature stop codon in front of the DNA-binding domain (Figure S5A). The *ets21c*^*Δ10*^ mutants are homozygous viable and progress to adulthood without noticeable developmental defects. To determine how the whole-body *ets21c* deficiency affects the intestinal epithelium, we used the *esg-GFP*^*YB0232*^ protein-trap line, which expresses GFP under the endogenous *esg* promoter (Kelso et al., 2004). The intestinal epithelium of 6-day-old *ets21c*^*Δ10*^ mutant adults was indistinguishable from control (*w*^*1118*^), comprising regularly organized GFP^+^ ISCs and EBs, Pros^+^ EEs, and polyploid ECs (Figures 6A and 6B). The midguts of 30-day-old *ets21c*^*Δ10*^ mutants did not show any signs of aging compared to the age-matched control guts, which displayed classical aging hallmarks, including accumulation of *esg*^*+*^ cells, immature pre-ECs, and aberrant tissue architecture (Figures 6C–6E). *Ets21c*^*Δ10*^ mutants thus recapitulated “rejuvenating” phenotypes observed upon *ets21c* knockdown in ISCs and ECs. Interestingly, both female and male *ets21c*^*Δ10*^ mutant flies lived significantly longer compared to control animals (Figures 6F and S5B–S5D). These data demonstrate that while *ets21c* is dispensable for normal development, its absence in adulthood prevents aging-associated tissue dysplasia and extends the lifespan.

In contrast, we found that flies lacking *ets21c* were more susceptible to paraquat toxicity and died significantly faster than control flies (Figures 6G and S5E). To determine whether the oxidative stress tolerance required Ets21c function in midgut progenitor cells or ECs, we repeated the stress experiments using the *esg*^*TS*^ and *Myo1A*^*TS*^ systems. Both inhibition and overexpression of *ets21c* in ISCs/EBs (*esg*^*TS*^ *> ets21c*^*RNAi*^ and *esg*^*TS*^ *> ets21c*^*WT*^) or in differentiated ECs (*Myo1A*^*TS*^ *> ets21c*^*RNAi*^ and *Myo1A*^*TS*^ *> ets21c*^*WT*^) had severe consequences for the survival of paraquat-exposed females (Figures 6H and 6I), while the effect on males was not significantly different from that on control animals, with the exception of *Myo1A*^*TS*^ *> ets21c*^*WT*^ males, which also died faster (Figures S5F and S5G). These results indicate that lack of Ets21c is beneficial under stress-free conditions, but it becomes deleterious upon exposure to stress or environmental challenge, when damaged cells must be quickly replenished to maintain tissue function. The resilience to stress requires balanced Ets21c activity in specific cell types of the intestine.

## Discussion

Here, we show that Ets21c, a member of the ETS-domain transcription factor family, functions as a critical regulator of intestinal tissue plasticity driving cellular responses to oxidative stress and aging in *Drosophila*. Our data demonstrate that Ets21c acts as a prime downstream effector of JNK signaling that is necessary and sufficient for the maintenance of proliferative homeostasis and epithelial turnover by orchestrating distinct cellular processes and cell-type-specific gene expression programs in progenitor and differentiated cells of the adult midgut.

By targeted manipulation of Ets21c in progenitor cells, we show that Ets21c levels affect the rate of ISC proliferation intrinsically while their maintenance and survival remain unaltered. The reduced ERK activation in ISCs as a consequence of *ets21c* deficiency in the stress-free context could provide a mechanism for the observed decline in ISC mitotic capacity. This would be consistent with a described dependency of the JNK-induced ISC hyperproliferation on the EGFR/Ras/ERK signaling pathway (Biteau and Jasper, 2011). The precise mechanism by which Ets21c regulates ERK activity and ISC proliferation remains to be determined. However, the identification of *pvf1* as a direct transcriptional target of Ets21c implies that Ets21c could modulate mitotic Pvr/Ras signaling in progenitors through the ISC-derived autocrine and EC-specific paracrine production of Pvf1 (Bond and Foley, 2012).

Differentiated ECs also require intrinsic Ets21c activity for proper function. We demonstrate that EC-specific Ets21c activation drives epithelial turnover, which involves the apoptotic removal of mature ECs and ISC proliferation to renew the pool of differentiated cells. We show that the Ets21c-mediated EC removal and compensatory ISC proliferation response could be suppressed by both co-expression of the pan-caspase inhibitor p35 or knockdown of the Ets21c target *eip93F* that controls Dcp1 activity. Neither *upd3* nor *pvf1* silencing in ECs interfered with Dcp1 activation, although both were indispensable for the non-autonomous induction of ISC proliferation by *ets21c-*expressing ECs. Based on these data, we conclude that Ets21c orchestrates epithelial turnover by promoting EC apoptosis and stimulating compensatory ISC proliferation by apoptosis-dependent and -independent mechanisms exploiting intercellular signaling molecules such as Pvf1 growth factor and the chief stress-inducible cytokine Upd3. Apoptosis-dependent and -independent induction of ISC proliferation has also been demonstrated for JNK signaling in ECs by Jiang et al. (2009). The cell death-independent mechanism would also explain why the ISC mitotic rate remains high in paraquat-exposed flies despite Dcp1 inactivation due to EC-specific *ets21c* silencing. In this respect, it is important to note that other transcription factors besides Ets21c have been shown to regulate Upd3 expression under stress conditions—for example, in infected ECs (Houtz et al., 2017) or upon oncogene activation in imaginal discs (Bunker et al., 2015, Külshammer et al., 2015).

Of note, increased JNK activity has been associated with age- and oxidative stress-related changes in the posterior midgut (Biteau et al., 2008, Choi et al., 2008). Consistent with its role as a JNK-dependent transcriptional effector, Ets21c levels build up in response to paraquat and during aging (our data Figures S1P and 2I; Guo et al., 2014). Our ISC/EB- and EC-specific TaDa profiling revealed that only a small fraction of the Ets21c-associated genes was actively transcribed, indicating that in the unstressed state, Ets21c contributes to the fine-tuning of gene expression that supports the steady-state epithelial replenishment. Its seemingly “unproductive” binding to DNA, however, likely primes a genetic program that can be rapidly executed in response to JNK activation upon challenge. This notion is supported by the significant enrichment for functional GO terms associated with stress-related JNK signaling. Furthermore, we show that Ets21c regulates genes that have been functionally linked to JNK signaling, including the *autophagy-related gene 1* (*atg1*) (Wu et al., 2009) and the *insulin signaling intersecting-target* (*Jafrac1*) (Biteau et al., 2010), or identified as JNK- and paraquat-responsive genes, such as the *eukaryotic translation initiation factor 2α kinase* (*PEK*), a *thioredoxin-like protein* (*fax*), or a *glutathione S-transferase* (*gstD10*) (Wang et al., 2003). We suggest that failure to accelerate intestinal regeneration and mount a robust cytoprotective response underlie the increased sensitivity of *ets21c*-deficient flies to oxidative stress. The capacity of Ets21c to confer cytoprotection but also trigger apoptosis is in line with the described roles of JNK signaling (Biteau et al., 2011b, Pinal et al., 2019). We propose that the repertoire of Ets21c-regulated target genes and the biological outcome of Ets21c activation depends on the strength and duration of cellular stress and the signaling landscape in which Ets21c operates. Such a model would be in accordance with the study from Loudhaief et al. (2017), showing that low stress levels can accelerate epithelial renewal in the absence of EC death due to moderate JNK induction that stimulates ISC division, while additional input from Hippo signaling accelerates apoptosis to prevent EC overcrowding.

In addition to its role in coordinating cellular behaviors within the intestine, Ets21c emerges as an important determinant of the adult intestinal healthspan and overall lifespan. Optimizing proliferative homeostasis in high-turnover tissues by, for example, moderate inhibition of insulin/IGF or JNK signaling activities has proven effective in prolonging the lifespan (Biteau et al., 2010). As Ets21c represents a key effector of JNK in the adult gut and its knockdown reduced ISC proliferation, it is plausible that balanced intestinal function may contribute to the lifespan extension of unchallenged *ets21c* mutant flies. However, the use of a full-body *ets21c* mutant prevents us from drawing a causal relation between the gut-specific role of Ets21c and longevity. The tissue- and cell-type-specific contribution of Ets21c to adult lifespan remains a question for future studies to address.

Finally, *ets21c* has been repeatedly picked up by gene expression profiling studies to be markedly increased in response to immune challenge, injury, oncogene activation, or aging. While functionally linked to JNK signaling in epithelial tumor models (Külshammer et al., 2015, Toggweiler et al., 2016), Ets21c has also been classified as an effector of EGFR/ERK signaling in the intestine based on the binding of Capicua, the EGFR/Ras/ERK-regulated transcriptional repressor, to the *ets21c* locus and upregulation of *ets21c* expression following Capicua loss (Jin et al., 2015). However, the functional evidence placing Ets21c downstream of EGFR/Ras/ERK signaling has been missing. Our data show that while knockdown of *ets21c* completely rescues the phenotypic consequences of JNK activation in the ISCs or ECs, it fails to mitigate the effects of activated EGFR/Ras/ERK signaling. Therefore, we propose that the regulation of *ets21c* levels results from an integration of positive and negative signaling inputs. This regulatory network includes Capicua, which acts as a gatekeeper of *ets21c* transcription. Such regulatory mechanisms ensure that JNK-Ets21c-mediated responses are fast but transient in supporting efficient tissue renewal while preventing chronic or excessive Ets21c activation, which drives tissue dysplasia and epithelial degeneration.

## STAR★Methods

### Key Resources Table

**Table:** 

REAGENT or RESOURCE	SOURCE	IDENTIFIER
**Antibodies**		

Mouse anti-Armadillo (Arm)	Developmental Studies Hybridoma Bank	DSHB #N27A1; RRID:AB_528089
Mouse anti-Prospero (Pros)	Developmental Studies Hybridoma Bank	DSHB #MR1A; RRID:AB_528440
Rabbit anti-cleaved Death caspase 1 (Dcp1)	Cell Signaling	Cat. #9578; RRID:AB_2721060
Rabbit anti-phospho-Histone H3 (pH3)	Cell Signaling	Cat. #9701; RRID:AB_331535
Rabbit anti-phospho-p44/42 MAPK (dpERK)	Cell Signaling	Cat. #4370; RRID:AB_2315112
Goat anti-GFP pAb	Abcam	Cat. #ab6673; RRID:AB_305643
Cy3 AP Donkey Anti-mouse IgG	Jackson ImmunoResearch	Cat. #715-165-151; RRID:AB_2315777
Cy3 AP Donkey Anti-rabbit IgG	Jackson ImmunoResearch	Cat. #711-165-152; RRID:AB_2307443
Cy5 AP Donkey Anti-mouse IgG	Jackson ImmunoResearch	Cat. #715-175-151; RRID:AB_2340820
Cy5 AP Donkey Anti-rabbit IgG	Jackson ImmunoResearch	Cat. #711-175-152; RRID:AB_2340607
Cy2 AP Donkey Anti-goat IgG (H+L)	Jackson ImmunoResearch	Cat. #705-225-147; RRID:AB_2307341

**Chemicals, Peptides, and Recombinant Proteins**		

4’,6-Diamidino-2-phenylindol Dihydrochlorid (DAPI)	Carl Roth GmbH	Cat. #6335
TRI Reagent	Sigma-Aldrich	Cat. #T9424
DNase I	Thermo Scientific	Cat. #AM2238
SuperScript III Reverse Transcriptase	Thermo Scientific	Cat. #18080044
GoTaq qPCR Master Mix	Promega	Cat. #A6000
DpnI	New England Biolabs	Cat. #R0176
T4 DNA Ligase (5 U/μl)	Roche	Cat. #799009
DpnII	New England Biolabs	Cat. #R0543
MyTaq HS DNA Polymerase	Bioline	Cat. #BIO-21112
AlwI	New England Biolabs	Cat. #R0513
Phusion High-Fidelity DNA Polymerase	New England Biolabs	Cat. #M0530
Paraquat	Sigma-Aldrich	Cat. #856177
1,4-Diazabicyclo[2.2.2]octane (DABCO)	Sigma-Aldrich	Cat. #D2522
Mowiol 4-88	Sigma-Aldrich	Cat. #81381

**Critical Commercial Assays**		

*In Situ* Cell Death Detection Kit, TMR red	Roche	Cat. #12156792910

**Deposited Data**		

Ets21c- and PolII-specific TaDa-seq from *esg*^*TS*^ and *Myo1A*^*TS*^ adult midgut samples (Raw and processed data)	This paper	GEO: GSE122170

**Experimental Models: Organisms/Strains**		

*w*^*1118*^	Bloomington *Drosophila* Stock Center	BDSC ID: 3605; RRID: BDSC_3605
*w; UAS-ets21c*^*RNAi*^	Vienna *Drosophila* Resource Center	VDRC ID: 106153
*w; UAS-ets21c*^*RNAi #2*^	This paper	N/A
*w; UAS-ets21c*^*WT*^	M. Uhlirova (Külshammer et al., 2015)	N/A
*w; UAS-hep*^*WT*^	D. Bohmann	BDSC ID: 9308; RRID: BDSC_9308
*w; UAS-ras*^*V12*^	T. Xu (Pagliarini and Xu, 2003)	N/A
*w; UAS-egfr*^*ACT*^	F. Hamaratoglu (Queenan et al., 1997)	N/A
*w; UAS-p35*	Bloomington *Drosophila* Stock Center	BDSC ID: 5072; RRID: BDSC_5072
*w; UAS-LT3-Dam*	A. H. Brand (Southall et al., 2013)	N/A
*w; UAS-LT3-Dam-Polymerase II*	A. H. Brand (Southall et al., 2013)	N/A
*w; UAS-LT3-Dam-ets21c-RA*	This paper	N/A
*w; UAS-imp*^*RNAi*^	Vienna *Drosophila* Resource Center	VDRC ID: 20322
*w; UAS-upd3*^*RNAi*^	Vienna *Drosophila* Resource Center	VDRC ID: 27136
*w; UAS-pvf1*^*RNAi*^	Vienna *Drosophila* Resource Center	VDRC ID: 102699
*w; UAS-eip93F*^*RNAi*^	Bloomington *Drosophila* Stock Center	BDSC ID: 57838; RRID: BDSC_57868
*w; esg-GFP*^*YB0232*^	L. O’Brien (Kelso et al., 2004)	N/A
*y w; esg-Gal4, UAS-GFP / CyO; tub-Gal80*^*TS*^	B. Biteau (Biteau et al., 2008)	N/A
*w; esg-Gal4, UAS-mCD8-GFP / CyO; UAS-H2B-RFP, tub-Gal80*^*TS*^*/ TM6B*	T. Reiff (Antonello et al., 2015)	N/A
*w; Myo1A-Gal4 / CyO; tub-Gal80*^*TS*^*, UAS-GFP / TM6B*	P. Patel (Jiang et al., 2009)	N/A
*y[1] M{vas-int.Dm}ZH-2A w^∗^;; P{CaryP}attP2*	L. Partridge	N/A
*y w Act5C-Cas9*	Bloomington *Drosophila* Stock Center	BDSC ID: 54590; RRID: BDSC_54590

**Oligonucleotides**		

Table S1	This paper	Table S1

**Recombinant DNA**		

*pWIZ*	*Drosophila* Genomics Resource Center (Lee and Carthew, 2003)	DGRC ID: 1008
*pWIZ-ets21c*^*RNAi #2*^	This study	N/A
*pUAST-attB-LT3-NDam*	A. H. Brand (Southall et al., 2013)	N/A
*pUAST-attB-LT3-Dam-ets21c-RA*	This paper	N/A

**Software and Algorithms**		

Fluoview 2.1c Software	Olympus	RRID:SCR_014215
Photoshop CS5.1	Adobe Systems, Inc.	RRID:SCR_014199
cellSens Standard 1.11 Software	Olympus	RRID:SCR_016238
Prism 5	GraphPad Software	RRID:SCR_002798
G^∗^Power Statistical Analysis	Faul et al., 2009	RRID:SCR_013726
Fiji	ImageJ	RRID:SCR_002285
Excel	Microsoft	RRID:SCR_016137
TaDa analysis scripts	T. Southall (Southall et al., 2013, Wolfram et al., 2012)	N/A
Gene Ontology	http://geneontology.org/	RRID:SCR_002811

**Other**		

CFX96 Real-Time PCR System	Bio-Rad	N/A
FV1000 Confocal Microscope	Olympus	N/A
CKX41 Inverted Microscope	Olympus	N/A
Active Motif EpiShear Sonicator	Active Motif	Cat. #53052
HiSeq 2000	Illumina, Inc.	N/A

### Contact for Reagent and Resource Sharing

Further information and requests for resources and reagents should be directed to and will be fulfilled by the Lead Contact, Mirka Uhlirova (mirka.uhlirova@uni-koeln.de).

### Experimental Model and Subject Details

#### *Drosophila melanogaster* Lines

The following *Drosophila melanogaster* strains were used: *w*^*1118*^, *UAS-ets21c*^*RNAi*^, *UAS-ets21c*^*RNAi #2*^, *UAS-ets21c*^*WT*^, *UAS-hep*^*WT*^, *UAS-ras*^*V12*^ (Pagliarini and Xu, 2003), *UAS-egfr*^*ACT*^ (Queenan et al., 1997), *UAS-p35*, *UAS-imp*^*RNAi*^, *UAS-upd3*^*RNAi*^, *UAS-pvf1*^*RNAi*^, *UAS-eip93F*^*RNAi*^, *esg-GFP*^*YB0232*^ (Kelso et al., 2004). For transgene expression, the following TARGET expression systems were used: *esg-Gal4, UAS-GFP, tub-Gal80*^*TS*^ (*esg*^*TS*^) (Biteau et al., 2008), *esg-Gal4, UAS-CD8-GFP, tub-Gal80*^*TS*^, *UAS-H2B-RFP* (*esg*^*TS*^*-ReDDM*) (Antonello et al., 2015), *Myo1A-Gal4, UAS-GFP, tub-Gal80*^*TS*^ (*Myo1A*^*TS*^) (Jiang et al., 2009).

*Ets21c*^*Δ10*^ mutant line was generated with the CRISPR/Cas9 method (Bassett et al., 2013) using a synthetic guide RNA against the first common exon of all annotated *ets21c* isoforms (sgRNA target sequence: GGATTTGGCCCCCTGAGCCT) (FlyBase Consortium, 2003), which was injected into *act5C-Cas9* embryos. Sequencing confirmed a 10 bp deletion resulting in a premature stop codon (Figure S5A). The *ets21c*^*Δ10*^ mutant stock was backcrossed to *w*^*1118*^ for ten generations.

An independent RNAi line against *ets21c* (*UAS-ets21c*^*RNAi #*2^) was generated by PCR amplification from cDNA using Phusion polymerase and the following primer combination: 5′- GGCGTGGTGATTGTAGGAAC-3′ and 5′- AACTACGACAAGCTGAGCCG-3′. Two fragments were cloned in anti-sense and sense orientation into the pWIZ vector (Lee and Carthew, 2003), enabling expression of RNA hairpin under UAS control. Transgenic flies were obtained by P-element mediated germline transformation (Genetic Services, Sudbury, USA).

#### *Drosophila* Husbandry

*Drosophila* stocks and experimental crosses were kept at room temperature on a diet consisting of 0.8% agar, 8% cornmeal, 1% soymeal, 1.8% dry yeast, 8% malt extract, and 2.2% sugar-beet syrup, which was supplemented with 0.625% propionic acid and 0.15% Nipagin. Experimental crosses were set up using virgins of the TARGET expression systems and males of the desired UAS transgenic lines or *w*^*1118*^ as control. For all experiments, adult offspring were collected for two days and mated for another two days at room temperature. Afterward adult flies were transferred into fly incubators with a 16 hours/8 hours light/dark cycle set to either 25°C (*w*^*1118*^ and *ets21c*^*Δ10*^ mutants) or 29°C (*esg*^*TS*^*, esg*^*TS*^*-ReDDM,* and *Myo1A*^*TS*^ TARGET expression systems) to age them for the indicated time. For recovery experiments, flies were kept for three days at 29°C and then shifted for another three days to 18°C.

### Method Details

#### Immunostainings

For all immunostainings female adult intestines of the indicated genotypes and age after temperature shift were dissected in PBS and fixed with 4% paraformaldehyde in PBS for 48 hours at 4°C to ensure thorough fixation to prevent gut deterioration. Primary antibodies were diluted in PBS with 0.3% BSA and 0.1% Triton X-100 and guts were stained overnight at 4°C. The following primary antibodies were used: Mouse anti-Armadillo (Arm, 1:20), mouse anti-Prospero (Pros, 1:20), rabbit anti-phospho-Histone H3 (pH3, 1:500), rabbit anti-cleaved Death caspase 1 (Dcp1, 1:200), rabbit anti-phospho-p44/42 MAPK (dpERK, 1:100). Guts were stained with corresponding secondary antibodies coupled to Cy3 or Cy5 (1:500) for two hours at room temperature and DAPI (1:1000 dilution of 5 mg/ml stock) was used to stain DNA. Intact guts were mounted in DABCO Mowiol 4-88 and imaged within 72 hours.

#### TUNNEL Assay

To detect apoptotic cells by TUNEL assay, female flies were dissected in PBS and fixed with 4% paraformaldehyde in PBS for 48 hours at 4°C. Guts were washed with PBST (PBS with 0.1% Triton X-100) and permeabilized for thirty minutes at room temperature with freshly prepared 0.1 M sodium citrate pH 6.0 mixed with 0.1% Triton X-100 in distilled water. Afterward guts were washed in distilled water. Samples dedicated to serve as a positive control (Figure S2E) were incubated with DNase I diluted 1:100 in distilled water for 15 minutes at room temperature. For each sample, 5 μl TdT enzyme solution were mixed with 45 μl label solution of the *In situ* Cell Death Detection Kit. Guts were incubated with the TUNEL reaction mix in the dark at 37°C for two hours. Guts were washed with PBST, blocked for one hour in the dark with PBST supplemented with 0.3% BSA and stained overnight at 4°C with primary goat anti-GFP antibody diluted 1:200 in PBST with 0.3% BSA. Guts were stained with the secondary anti-goat Cy2 for two hours at room temperature and DAPI was used to stain DNA. Guts mounted in DABCO Mowiol 4-88 were imaged on the same day.

#### Image Acquisition and Analysis

Confocal images and stacks were acquired with Olympus FV1000 confocal microscope equipped with 20x UPlan S-Apo (NA 0.85), 40x UPlan FL (NA 1.30) and 60x UPlanApo (NA 1.35) objectives. Images were always taken from the R5 posterior midgut region. All micrographs show maximum projections generated with Fluoview 2.1c Software, except panels showing midgut cross-sections (Figures 3I–3K) which are single sections. Final image processing including panel assembly, brightness, and contrast adjustment were done in Photoshop CS5.5.

The mitotic indices were evaluated by a single person in a non-blinded fashion by manually counting all pH3-positive cells in intact midguts of female adult flies of indicated genotypes and conditions using an inverted fluorescent Olympus CKX41 microscope.

To measure the tube diameter, bright field images of female adult midguts were taken with the cellSens Standard 1.11 software using an Olympus CKX41 inverted microscope and imported into Photoshop CS5.5. Measurements were taken at a distance of 200 μm anterior to the midgut-hindgut boundary for each gut from outside the visceral mesoderm surrounding the midgut.

For cell type counts, image stacks were generated with an Olympus FV1000 confocal microscope from a 100 μm x 100 μm field in the R5 posterior midgut region (Buchon et al., 2013). Cells were manually counted by a single person in a non-blinded fashion in Fiji based on DAPI and three GFP intensity levels.

#### Lifespan and Survival Experiments

For lifespan experiments, mated male and female flies were separated and transferred into 1 L cages (75-100 flies/cage) with access to a vial containing the standard fly diet. Dead flies were counted and the food vial was exchanged every two to three days.

For stress experiments, empty vials were prepared with a filter paper containing either 500 μl 5% sucrose (Mock) or 500 μl 5 mM paraquat diluted in 5% sucrose (PQ). Each vial contained twenty flies separated according to their sex. Mortality was scored every 4-8 hours by a single person in a non-blinded fashion. Fresh 200 μl of the respective solution was added to the filter papers every 24 hours. For confocal microscopy, female guts were dissected 24 hours following paraquat feeding.

#### Gene Expression Analysis

For each biological replicate of indicated genotype and condition, total RNA was isolated from 15-20 female adult midguts using the standard protocol with TRI Reagent and DNase I treatment (Mundorf and Uhlirova, 2016). cDNA was synthesized from 1-2 μg of RNA using oligo(dT) primers and SuperScript III Reverse Transcriptase. Quantitative PCR (RT-qPCR) was performed in triplicates with GoTaq qPCR Master Mix in the CFX96 real-time PCR system. The mRNA expression of indicated genes (see Table S1 for primer sequences) was normalized to the levels of *rp49* transcript and fold changes were calculated using the ΔΔCT method (Livak and Schmittgen, 2001).

#### *In vivo* Targeted DamID and Data Analysis

The coding sequence of *ets21c-RA* isoform was amplified from cDNA and cloned into the pUAST-attB-LT3-NDam vector to allow temporarily and spatially inducible expression with low-level translation (Southall et al., 2013). The transgenic *ets21c*^*DAM*^ fly line was obtained by inserting *UAS-LT3-Dam-ets21c-RA* construct into the attP2 landing site which was also used for the control fly lines *UAS-LT3-Dam* and *UAS-LT3-Dam-PolII* (*PolII*^*DAM*^) (Southall et al., 2013). Males of *UAS-LT3-Dam, UAS-LT3-Dam-PolII* and *UAS-LT3-Dam-ets21c-RA* stocks were crossed to *esg*^*TS*^ and *Myo1A*^*TS*^ virgins and kept at room temperature. Offspring collected for two days were mated for another two days at room temperature. Twenty females per vial were transferred to permissive temperature (29°C) and aged for three days before midgut dissection.

The TaDa protocol was performed as described in Vogel et al. (2007) with minor modifications. Genomic DNA was extracted by phenol-chloroform extraction and ethanol precipitation from whole midguts without proventriculus using either 30 (*Myo1A*^*TS*^) or 50 (*esg*^*TS*^) midguts for each of the three biological replicates per genotype. After DpnI digestion, adaptor ligation and DpnII digestion methylated sequences were amplified with the MyTaq HS DNA polymerase. PCR products were prepared for sequencing following the protocol of Marshall et al. (2016) with minor modifications. PCR products extracted with phenol-chloroform were sheared with an Active Motif EpiShear Sonicator to obtain fragments of a 300 bp mean size and digested with AlwI to remove the adapters. Sequencing libraries generated according to the Illumina protocol for ChIP-Seq library preparation were single-end sequenced on an Illumina HiSeq 2000 instrument at 50 bp read length.

Sequencing data were processed using the damidseq_pipeline (Marshall and Brand, 2015) to generate gff files containing the normalized log2 ratio (Dam-fusion/Dam) per GATC fragment. *Myo1A*^*TS*^ *> ets21c*^*DAM*^ (replicate two) and *esg*^*TS*^ *> ets21c*^*DAM*^ (replicate two) data were noisy compared to replicates one and three, therefore were omitted from further analysis.

Ets21c binding peaks were identified using a peak finding program based on one described by Wolfram et al. (2012). Each replicate dataset was analyzed separately: A false discovery rate (FDR) was calculated for peaks (formed of two or more consecutive GATC fragments) for the individual replicates. Then each potential peak in the data was assigned a FDR. Any peaks with less than a 1% FDR were classified as significant. Additionally, a mean log2 ratio threshold of 0.3 for each peak was implemented. Significant peaks present in all replicates were used to form a final peak file. Any gene within 5 kb of a peak (with no other genes in between) was identified as a potential target.

Transcribed genes (defined by PolII occupancy) were identified using a Perl script based on one described by Southall et al. (2013). Each replicate dataset was analyzed separately. Using *Drosophila* genome annotation release 6.03 (dm6), the mean ratio changes across each annotated transcript were calculated. To assign a FDR value, the frequency of transcripts with a mean ratio over specific values (ranging from 0 to 0.75 log2 increase) were calculated within a randomized dataset (for each chromosome arm) using 10 iterations and 1,000 sampling events. This was repeated for a range of gene sizes (250 to 2,500 bp). These data were used to model FDR values relative to the PolII^DAM^ enrichment across a transcript and gene sizes, therefore enabling extrapolation of FDR values for larger ratio changes and larger transcripts. After being performed for each replicate, each transcript was assigned a mean ratio between the biological replicates and the highest associated FDR.

Gene ontology (GO) cluster analysis was performed using the geneontology.org webpage by uploading the top 250 Ets21c-bound genes either transcribed (PolII-bound) or non-transcribed (no PolII-binding) (see also Table S3). For visualization, GO terms were manually grouped into indicated GO clusters (see also Table S3) and GraphPad Prism was used to prepare scattered plots.

### Quantification and Statistical Analysis

Scattered plots of pH3^+^ cell quantifications (related to Figures 1F, 1J, 1O, 1S, S1O, 2A, 3N, and 5P) show the means with standard deviation (SD) combining at least two independent experiments. Each dot represents the number of dividing cells per single midgut and the number of total midguts (n) is specified in the respective figure legends. Statistical significance was determined in GraphPad Prism using an unpaired two-tailed Student’s t test with unequal variance: ^∗^p < 0.05, ^∗∗^p < 0.01, ^∗∗∗^p < 0.001, n.s. = non-significant. Post hoc analysis of the pH3 counts using G^∗^Power test determined that the statistical power of all significant differences (1-beta) exceeded 0.99, given the respective sample sizes, means, and standard deviations (Faul et al., 2009).

Scattered plots of posterior midgut diameter measurements (related to Figures 3E and S1S) show the means with standard deviation with each dot representing the diameter of a single midgut in micrometers. The number of analyzed midguts (n) is specified in the respective figure legends. Statistical significance was determined in GraphPad Prism using an unpaired two-tailed Student’s t test with unequal variance: ^∗^p < 0.05, ^∗∗^p < 0.01, ^∗∗∗^p < 0.001, n.s. = non-significant.

Stacked bar graphs of cell type quantifications (related to Figure 6E) were generated and statistical significance was determined by a Two-way ANOVA test in GraphPad Prism: ^∗∗∗^p < 0.0001. The total number of evaluated guts (n) is specified in the respective figure legend.

Adult fly lifespan and survival were analyzed in Microsoft Excel and statistical significance among genotypes was calculated with a chi-square log-rank test: ^∗^p < 0.05, ^∗∗^p < 0.01, ^∗∗∗^p < 0.001, n.s. = non-significant. The curves prepared with GraphPad Prism represent one of two to three independent experiments. The total number of flies per experiment, genotype, and condition (n) is specified in the respective figure legends.

The RT-qPCR data are presented either as scattered plots (related to Figure 1B, S1P, and 2I) or column bar graphs (related to Figure S3C and 4C). An unpaired two-tailed Student’s t test with unequal variance was used to determine statistical significance in GraphPad Prism: ^∗^p < 0.05, ^∗∗^p < 0.01, ^∗∗∗^p < 0.001, n.s. = non-significant. The number of biological replicates (n) is specified in the respective figure legends. Post hoc analysis of RT-qPCR data using G^∗^Power test determined that the statistical power of all significant differences (1-beta) exceeded 0.90, given the respective sample sizes, means, and standard deviations.

For raw data, quantifications, and statistical analyses see Table S5.

### Data and Software Availability

The TaDa sequencing data reported in this paper have been deposited in the Gene Expression Omnibus (GEO) database under the accession number GEO: GSE122170. Scripts for TaDa analysis are available on request from T.D.S.

## Supplemental Information

Document S1. Figures S1–S5 and Table S1

Table S2. Ets21c and PolII TaDa-Sequencing (TaDa-Seq) Results, Related to Figures 4 and S2

Table S3. GO Cluster Analysis, Related to Table S2 and Figures 4 and S3

Table S4. TaDa-Seq Comparison to Published Genome-wide Sequencing Datasets, Related to Table S2 and Figure 4

Table S5. Raw Data, Quantifications, and Statistical Analysis, Related to Figures 1, 2, 3, 4, 5, 6, S1, S3, S4, and S5

Document S2. Article plus Supplemental Information
