# Supplementary material for: Ets21c Governs Tissue Renewal, Stress Tolerance, and Aging in the Drosophila Intestine
Source: Cell Rep. 2019 Jun 4;27(10):3019–3033.e5. doi: 10.1016/j.celrep.2019.05.025 (PMC6581828; doi:10.1016/j.celrep.2019.05.025)
Supplement: Document S1. Figures S1–S5 and Table S1 [file mmc1.pdf]

Cell Reports, Volume *27*

Supplemental Information

Ets21c Governs Tissue Renewal, Stress Tolerance, and Aging in the *Drosophila* Intestine

Juliane Mundorf, Colin D. Donohoe, Colin D. McClure, Tony D. Southall, and Mirka Uhlirova

Supplemental Information

Figure S1. Ets21c is not required for EGFR/ERK signaling functions in the intestine. Related to Figure 1.

Figure S2. Ets21c triggers EC apoptosis. Related to Figure 3.

Figure S3. Ets21c binds to actively transcribed genes as well as those devoid of PolII binding. Related to Figure 4.

Figure S4. Cell type-specific sets of target genes mediate the cellular responses to Ets21c. Related to Figure 5.

Figure S5. ***Ets21c***$^{\Delta 10}$ **mutants live longer but have reduced stress tolerance.** Related to Figure 6.

Table S1. Primers. Related to Key Resources Table.

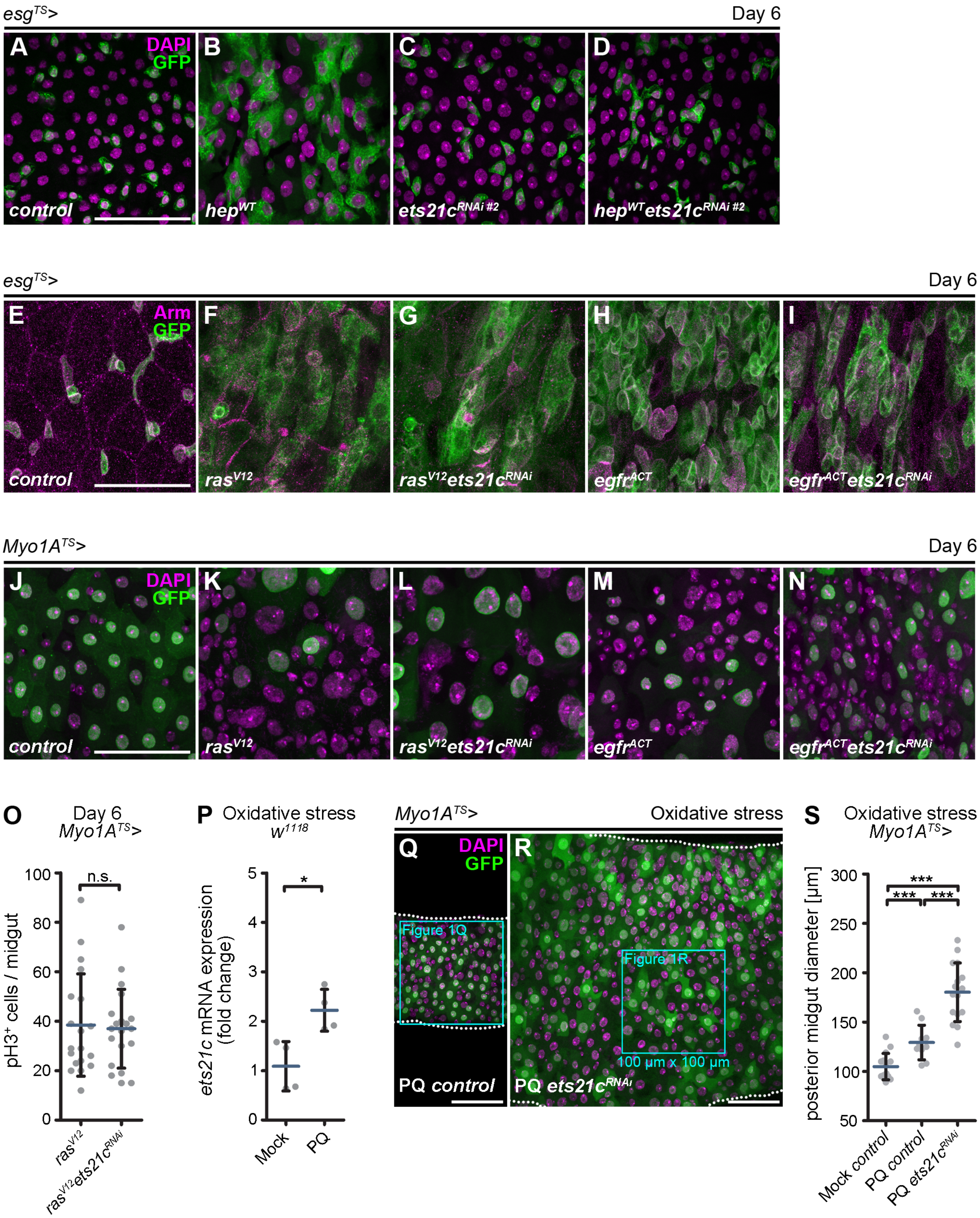

esgTS>
Day 6
DAPI
GFP
A control
B hepWT
C ets21cRNAi #2
D hepWTets21cRNAi #2
esgTS>
Day 6
Arm
GFP
E control
F rasV12
G rasV12ets21cRNAi
H egfrACT
I egfrACTets21cRNAi
Myo1ATS>
Day 6
DAPI
GFP
J control
K rasV12
L rasV12ets21cRNAi
M egfrACT
N egfrACTets21cRNAi
O Day 6 Myo1ATS>
pH3+ cells / midgut
n.s.
rasV12
rasV12ets21cRNAi
P Oxidative stress w1118
ets21c mRNA expression (fold change)
*
Mock
PQ
Myo1ATS>
Oxidative stress
Q DAPI GFP
Figure 1Q
PQ control
R
Figure 1R
100 µm x 100 µm
PQ ets21cRNAi
S Oxidative stress Myo1ATS>
posterior midgut diameter [µm]
***
Mock control
PQ control
PQ ets21cRNAi

Figure S1. Ets21c is not required for EGFR/ERK signaling functions in the intestine.
Related to Figure 1.

(A-D) Compared to *esg*^{TS} control midguts (A), JNK activation (*esg*^{TS}>*hep*^{WT}) for six days induced expansion of *esg*-expressing cells (B). JNK-induced gut dysplasia was suppressed by silencing *ets21c* with an independent RNAi line (*esg*^{TS}>*hep*^{WT}*ets21c*$^{RNAi\ \#2}$) (D) which by itself did not impact ISCs/EBs (C). **(E-I)** Compared to *esg*^{TS} control posterior midguts (E), overexpression of a constitutively active *ras*^{V12} (F) or *egfr*^{ACT} (H) in ISCs/EBs for six days caused accumulation of GFP-positive cells and intestinal dysplasia in Ets21c-independent manner (G, I). **(J-N)** Compared to six-day-old *Myo1A*^{TS} control midguts (J), the loss of GFP-expressing ECs and excessive endoreplication of remaining *Myo1A*-positive cells caused by EC-specific hyperactivation of EGFR/ERK signaling by Ras^{V12} (K) or EGFR^{ACT} (M) was not alleviated by silencing *ets21c* (L, N). **(O)** Ets21c (*Myo1A*^{TS}>*ras*^{V12}*ets21c*^{RNAi}) appeared dispensable for ISC proliferation response (pH3^{+} cells) induced by EC-specific EGFR/ERK activation (*Myo1A*^{TS}>*ras*^{V12}). Data represent means (SD), n=20; n.s. = non-significant. **(P)** *ets21c* expression increased in midguts of female *w*^{1118} flies fed with 5 mM paraquat (PQ) for 24 hours relative to unstressed controls (Mock). RT-qPCR data represent means (SD), n=4; *P<0.05. **(Q-S)** Compared to *Myo1A*^{TS} control midguts of flies exposed to 5 mM PQ for 24 hours (Q), midguts with EC-specific *ets21c* knockdown (*Myo1A*^{TS}>*ets21c*^{RNAi}) accumulated GFP-expressing cells (R) and their diameter dramatically increased (S) relative to mock- and PQ-treated controls. (Q, R) Blue outlines represent frames used for confocal images in Figure 1Q and 1R, respectively. (S) Data represent means (SD), n=10-17; ***P<0.001. (A-N, Q, R) Images are projections of multiple confocal sections taken from the R5 posterior midgut region. Nuclei were stained with DAPI. Scale bars: 50 µm. See also Figure 1.

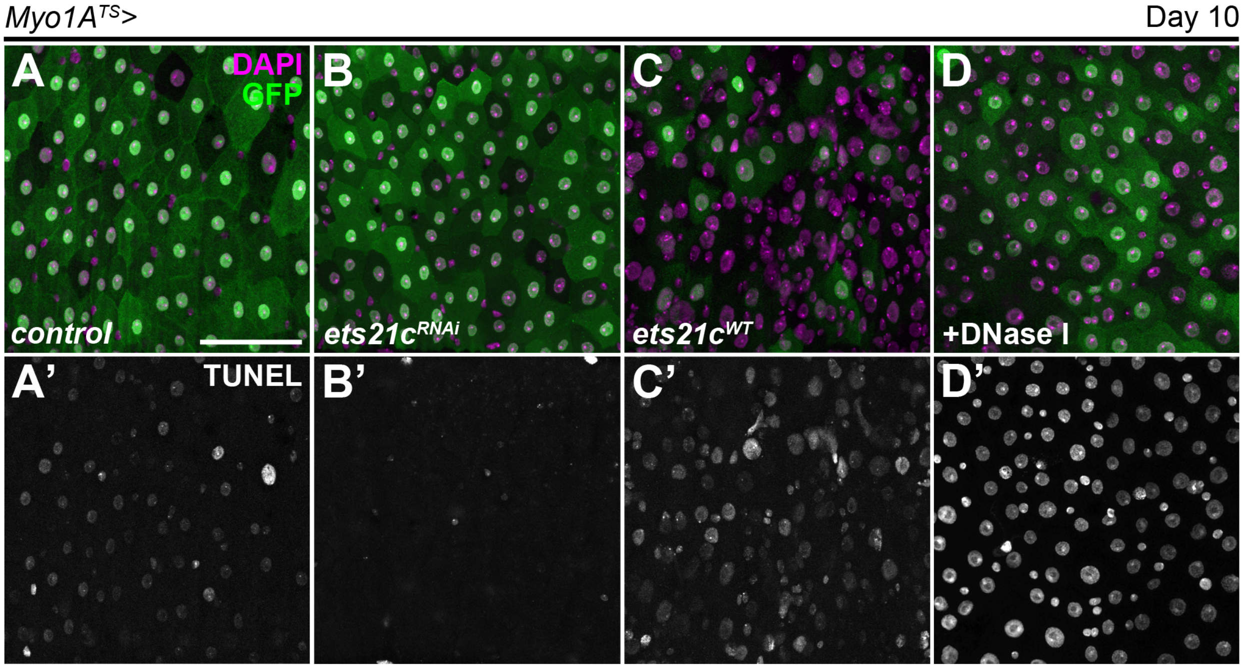

Figure S2. Ets21c triggers EC apoptosis. Related to Figure 3.

(A-D) Compared to ten-day-old *Myo1A*^{TS} control posterior midguts (A), EC-specific knockdown of *ets21c* (*Myo1A*^{TS}*>ets21c*^{RNAi}) suppressed EC apoptosis marked by TUNEL assay (B) while *ets21c* overexpression (*Myo1A*^{TS}*>ets21c*^{WT}) enhanced it (C). DNase I treatment of midguts was included as positive control for the TUNEL assay (D). Images are projections of multiple confocal sections taken from the R5 posterior midgut region. Nuclei were stained with DAPI. Scale bar: 50 µm. See also Figure 3.

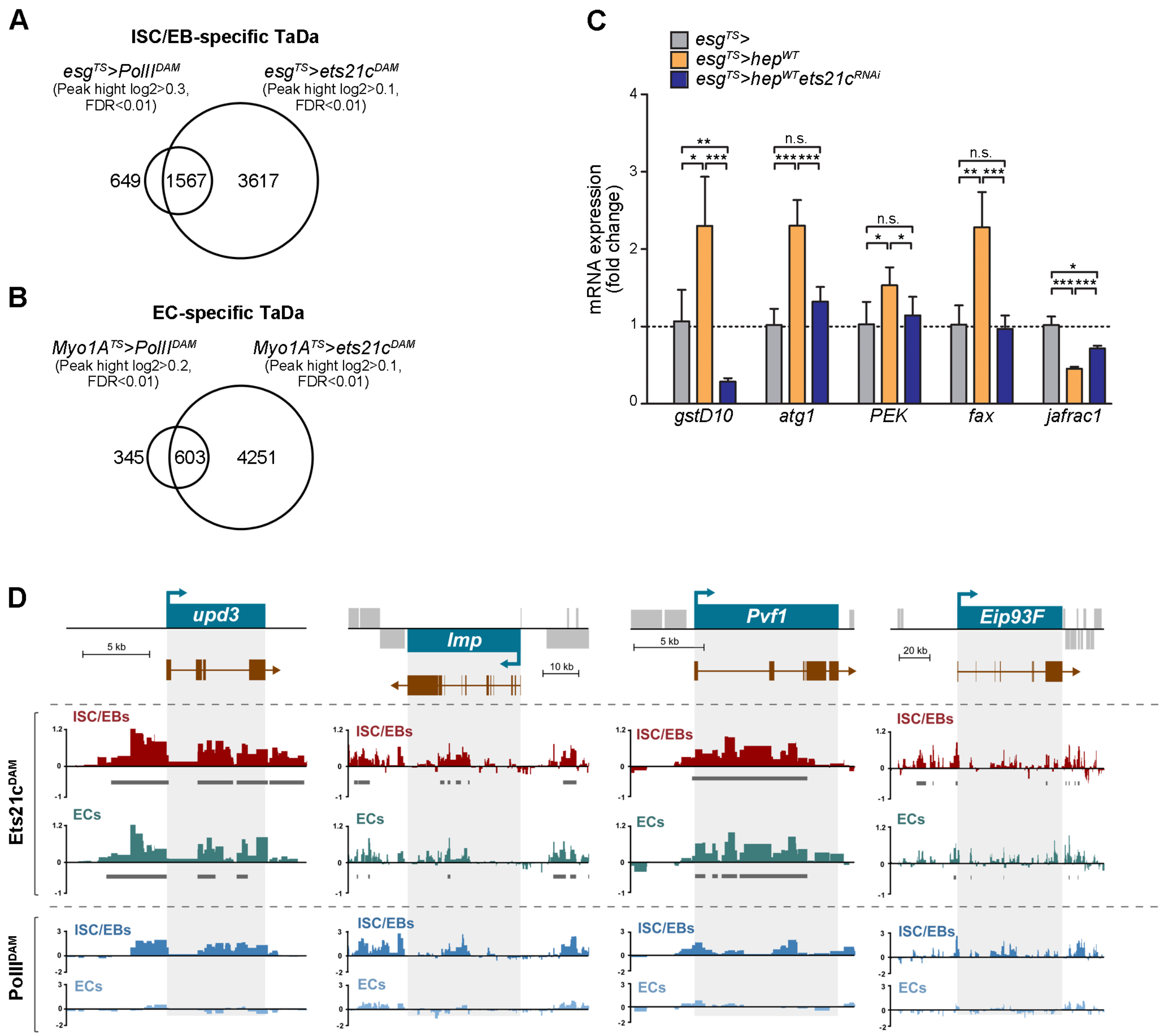

Figure S3. Ets21c binds to actively transcribed genes as well as those devoid of PolII binding. Related to Figure 4.

(A-B) The Venn diagrams show overlaps of genes identified by a TaDa approach that are actively transcribed ($PolII^{DAM}$) and bound by Ets21c ($Ets21c^{DAM}$) in either ISCs/EBs or ECs using the esg^{TS} (A) or $Myo1A^{TS}$ system (B) (see also Table S2). **(C)** JNK activation in progenitor cells ($esg^{TS}>hep^{WT}$) induced mRNA expression of selected cytoprotective, autophagy-related, and IIS-associated genes in an Ets21c-dependent manner ($esg^{TS}>hep^{WT}ets21c^{RNAi}$). RT-qPCR data represent means (SD), n=4-5; *$P<0.05$, **$P<0.01$, ***$P<0.001$, n.s. = non-significant. **(D)** Profiles of Ets21c and PolII occupancy in the gene loci of putative Ets21c target genes identified by esg^{TS}- and $Myo1A^{TS}$-specific TaDa approach. Y-axis represents log2 ratios of $Ets21c^{DAM}$- and $PolII^{DAM}$-specific sequencing peaks compared to the Dam-only control. Grey lines below the tracks depict regions of significant Ets21c binding. See also Figure 4 and Table S2.

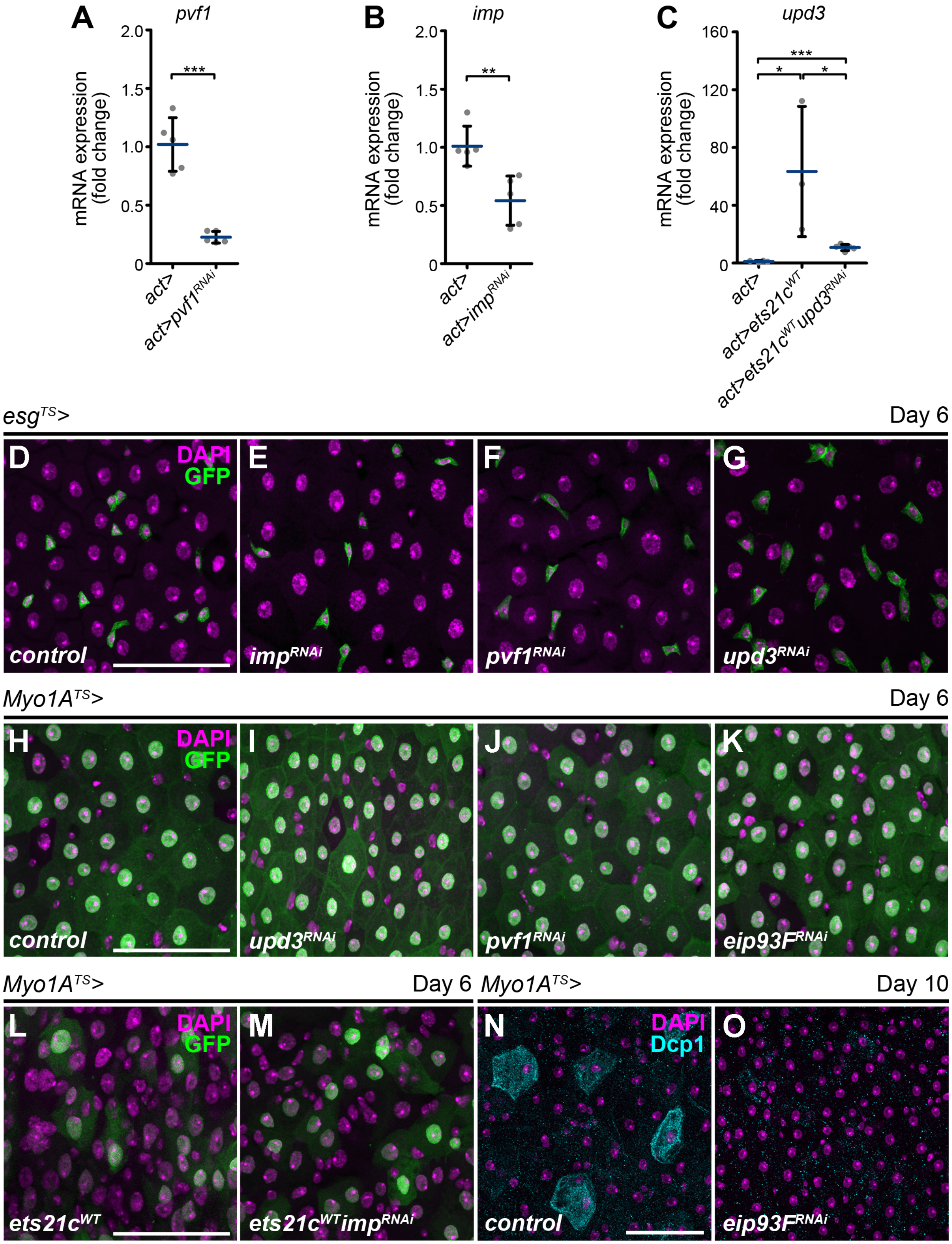

A
pvf1
mRNA expression (fold change)
2.0
1.5
1.0
0.5
0
***
act>
act>pvf1RNAi
B
imp
mRNA expression (fold change)
2.0
1.5
1.0
0.5
0
**
act>
act>impRNAi
C
upd3
mRNA expression (fold change)
160
120
80
40
0
***
*
*
act>
act>ets21cWT
act>ets21cWT upd3RNAi
esgTS>
Day 6
D
DAPI
GFP
control
E
impRNAi
F
pvf1RNAi
G
upd3RNAi
Myo1ATS>
Day 6
H
DAPI
GFP
control
I
upd3RNAi
J
pvf1RNAi
K
eip93FRNAi
Myo1ATS>
Day 6
Myo1ATS>
Day 10
L
DAPI
GFP
ets21cWT
M
ets21cWTimpRNAi
N
DAPI
Dcp1
control
O
eip93FRNAi

Figure S4. Cell type-specific sets of target genes mediate the cellular responses to Ets21c. Related to Figure 5.

(A-C) Ubiquitous expression of *pvf1*^{RNAi} (A), *imp*^{RNAi} (B), and *upd3*^{RNAi} (C) transgenic lines using the *actin-Gal4 (act>)* driver significantly downregulated the respective transcripts. Due to low levels of *upd3* mRNA in unstressed animals, *ets21c* was co-expressed (*act>ets21c*^{WT}) to enhance *upd3* transcription. RNA was isolated from the third instar larvae. RT-qPCR data represent means (SD), n=3-5; *P<0.05, **P<0.01, ***P<0.001. **(D-G)** ISC/EB-specific knockdown of predicted Ets21c target genes using the *esg*^{TS} system (D) showed that neither *imp* (E), *pvf1* (F), nor *upd3* (G) had an impact on progenitor cells in young unstressed flies. **(H-K)** EC-specific knockdown of *upd3* (I), *pvf1* (J), and *eip93F* (K) using the *Myo1A*^{TS} system resulted in control-like intestinal epithelia (H). **(L-M)** Ets21c-induced loss of GFP-expressing ECs (*Myo1A*^{TS}*>ets21c*^{WT}) (L) was not suppressed by *imp* silencing (*Myo1A*^{TS}*>ets21c*^{WT}*imp*^{RNAi}) (M). **(N-O)** Compared to a Dcp1 enrichment in several ECs of ten-day-old *Myo1A*^{TS} control posterior midguts (N), blocking Eip93F in ECs (*Myo1A*^{TS}*>eip93F*^{RNAi}) completely abolished Dcp1 activation (O). (D-O) Images are projections of multiple confocal sections taken from the R5 posterior midgut region. Nuclei were stained with DAPI. Scale bars: 50 µm. See also Figure 5.

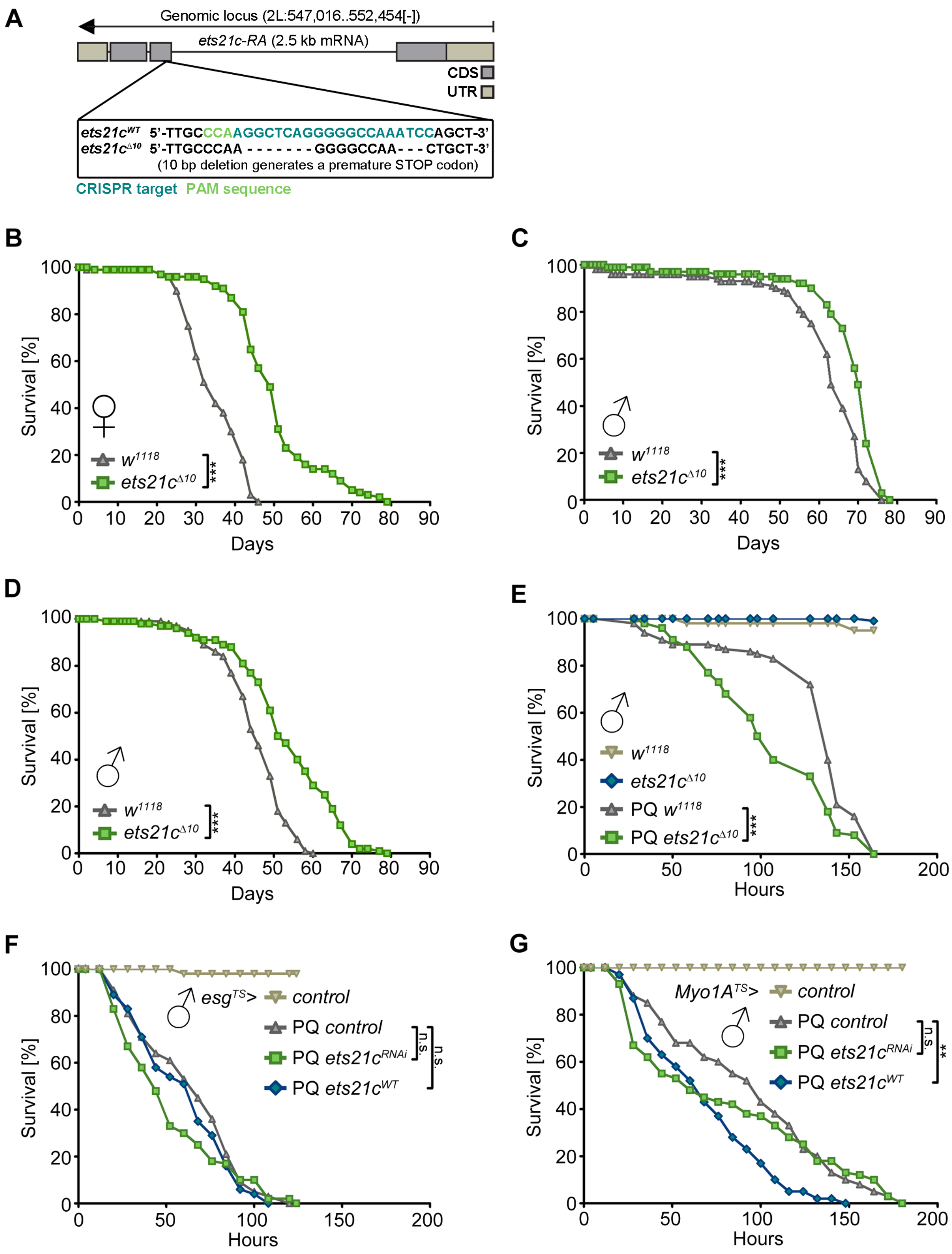
A
Genomic locus (2L:547,016..552,454[-])
ets21c-RA (2.5 kb mRNA)
CDS
UTR
ets21cWT 5'-TTGCCCAAGGCTCAGGGGGCCAAATCCAGCT-3'
ets21cΔ10 5'-TTGCCCAA - - - - - - - GGGGCCAA- - - CTGCT-3'
(10 bp deletion generates a premature STOP codon)
CRISPR target PAM sequence
B
Survival [%]
Days
w1118
ets21cΔ10
***
C
Survival [%]
Days
w1118
ets21cΔ10
***
D
Survival [%]
Days
w1118
ets21cΔ10
***
E
Survival [%]
Hours
w1118
ets21cΔ10
PQ w1118
PQ ets21cΔ10
***
F
Survival [%]
Hours
esgTS>
control
PQ control
PQ ets21cRNAi
PQ ets21cWT
n.s.
n.s.
G
Survival [%]
Hours
Myo1ATS>
control
PQ control
PQ ets21cRNAi
PQ ets21cWT
n.s.
**

Figure S5. ***Ets21c$^{\Delta 10}$*** **mutants live longer but have reduced stress tolerance.** Related to Figure 6.

(A) *Ets21c$^{\Delta 10}$* mutants generated by CRISPR-Cas9 contain a 10 bp deletion in the *ets21c* open reading frame, which results in a premature stop codon upstream of the ETS DNA-binding domain. CDS, coding sequence; UTR, untranslated region; PAM, protospacer adjacent motif. **(B)** The *ets21c$^{\Delta 10}$* homozygous mutant females (n=383) lived significantly longer compared to *w^{1118}* control females (n=425) (mean difference of 10 days). **(C)** Compared to *w^{1118}* control male flies (n=199), *ets21c$^{\Delta 10}$* homozygous mutants (n=146) had extended lifespan (mean difference of 4 days). **(D)** Homozygous *ets21c$^{\Delta 10}$* mutants (n=392) lived longer compared to *w^{1118}* control male flies (n=420) (mean difference of 3 days). **(E)** Feeding adult males with 5 mM paraquat (PQ) compared to mock solution resulted in premature death of *ets21c$^{\Delta 10}$* mutants (Mock n=80; PQ n=120) compared to *w^{1118}* control flies (Mock n=40; PQ n=100) (mean difference of 24 hours). **(F)** ISC/EB-specific Ets21c inhibition (*esg^{TS}>ets21c^{RNAi}*; n=80; mean difference of 10 hours) and overexpression (*esg^{TS}>ets21c^{WT}*; n=60; mean difference of 2 hours) had negligible effects on fly survival compared to *esg^{TS}* control males (n=80) following paraquat exposure. **(G)** Compared to *Myo1A^{TS}* control males (n=60), silencing of *ets21c* had no significant effect (*Myo1A^{TS}>ets21c^{RNAi}*; n=60; mean difference of 19 hours) while ectopic *ets21c* expression in ECs provoked premature death (*Myo1A^{TS}>ets21c^{WT}*; n=60; mean difference of 10 hours) of flies fed with paraquat. (B-G) Lifespan and survival curves represent one of the two to three independent experiments. Statistical significance was determined by Log Rank test; $^{**}P<0.01$, $^{***}P<0.001$, n.s. = non-significant. See also Figure 6.

Table S1. Primers. Related to Key Resources Table.

Gene	Application	Related to	Primer sequence
atg1	RT-qPCR	Figure S3C	5'-TATTGCCGCTTCGACGCAAC-3'<br>5'-CAGCCAATTAGCGTAAAGCAAC-3'
eip93F	RT-qPCR	Figure 4C	5'-TGCAACTTCTGTGTTAACGGTCGC-3'<br>5'-GCCACTGCTATTGTTGTTGCTGCT-3'
ets21c	RT-qPCR	Figure S1P and 2I	5'-ATTAATGCCATGCATCAGGATGTCCG-3'<br>5'-GTGGGAACTTCCGTCTCCTTCG-3'
ets21c (#2)	RT-qPCR	Figure 1B	5'-GAATACGGCGCTACTCTTAACC-3'<br>5'-GATGATTCACCCGAGATAGTCAG-3'
ets21c$^{RNAi\ \#2}$	RNAi cloning	Figure S1C-D	5'-GGCGTGGTGATTGTAGGAAC-3'<br>5'-AACTACGACAAGCTGAGCCG-3'
fax	RT-qPCR	Figure S3C	5'-GCAAGGACGACCTGAAGGTG-3'<br>5'-CATTGAGGTCCAGCTTCGTG-3'
gstD10	RT-qPCR	Figure S3C	5'-GAAGACCATTATCAACACCCG-3'<br>5'-CTCTTATACAGCGTACCCATG-3'
imp	RT-qPCR	Figure 4C and S4B	5'-CGTAGCCAGCGTAACCAGCG-3'<br>5'-CTCCAGCGATCCAACATTCTC-3'
jafrac1	RT-qPCR	Figure S3C	5'-GACATCAAGTTGAGCGACTAC-3'<br>5'-CCACCTTCATCGACTTGTCAG-3'
PEK	RT-qPCR	Figure S3C	5'-CAGTTCGTACGCATGATTCCC-3'<br>5'-CTCATCGGGCTCCCTAATGG-3'
pvf1	RT-qPCR	Figure 4C and S4A	5'-CGCCACGGAGTACGAAGTAG-3'<br>5'-GGCAAATATCGAATCGTCAGAG-3'
rp49	RT-qPCR	Figure 1B, 2I, 4C,<br>S1P, S3C, and S4A-C	5'- TCCTACCAGCTTCAAGATGAC-3'<br>5'-CACGTTGTGCACCAGGAACT-3'
upd3	RT-qPCR	Figure 4C and S4C	5'-AGTGAGCACCAAGACTCTGGACAT-3'<br>5'-GTGGCGAAGGTTCAACTGTTTGCT-3'
